# Comparative effectiveness of non-pharmacological traditional Chinese medicine therapies for chronic fatigue syndrome: a systematic review and network meta-analysis

**DOI:** 10.3389/fmed.2026.1804710

**Published:** 2026-04-01

**Authors:** Yihan Zhang, You Zhou, Hangying Xu, Chenxin Zhang, Lingyi Guo, Aiqun Zi, Yan Xu, Min Xu, Ting Liu

**Affiliations:** 1 School of Nursing, Zhejiang Chinese Medical University, Hangzhou, Zhejiang, China; 2 Department of Nursing, The First Affiliated Hospital of Zhejiang Chinese Medical University (Zhejiang Provincial Hospital of Chinese Medicine), Hangzhou, Zhejiang, China

**Keywords:** complementary therapies, fatigue syndrome, chronic, medicine, Chinese traditional, network meta-analysis, systematic review

## Abstract

**Background:**

Chronic fatigue syndrome (CFS) is a systemic disorder with symptoms, including persistent fatigue, unrefreshing sleep, anxiety, and depression, raising health concerns. The effectiveness of non-pharmacological Traditional Chinese Medicine (TCM) therapies for CFS has not been systematically investigated.

**Objective:**

This study was designed to investigate the comparative effectiveness of non-pharmacological TCM therapies for CFS.

**Methods:**

Searches of Web of Science, Embase, PubMed, Scopus, CINHAL, Cochrane Library, CNKI, WanFang, VIP database, and SinoMed were conducted on January 1, 2026. The Cochrane Risk of Bias Assessment Tool and CINeMA web application were used to assess the risk of bias. Pairwise meta-analysis and NMA were performed using Stata 18.0.

**Results:**

A total of 29 studies involving 2,234 participants were included in the analysis. Compared with the conventional care, moxibustion was associated with a reduction in overall fatigue (SMD, −1.84; 95% CI, −2.25 to −1.44). Massage was associated with reduced physical fatigue (SMD, −2.21; 95% CI, −2.65 to −1.77), mental fatigue (SMD, −2.05; 95% CI, −3.07 to −1.02), anxiety (SMD, −3.35; 95% CI, −6.64 to −0.05) and depression (SMD, −1.23; 95% CI, −1.76 to −0.69) vs. conventional care. Cupping therapy showed the greatest improvement in sleep quality (SMD, −4.60; 95% CI, −7.05 to −2.15) vs. conventional care.

**Conclusion:**

Current evidence suggests that within the non-pharmacological TCM clinical framework, moxibustion is more effective for overall fatigue. Massage better alleviated physical and mental fatigue, anxiety, and depression, while cupping improved sleep quality. These findings suggest that different non-pharmacological TCM therapies may have varying effects across multiple symptom areas. However, the results should be interpreted with caution, given the uncertainties in the available evidence and the limitations of the included studies.

**Systematic review registration:**

https://www.crd.york.ac.uk/PROSPERO/, Identifier CRD420251113292.

## 1 Introduction

Chronic fatigue syndrome (CFS) is a chronic, systemic disorder with a complex etiology, affecting an estimated 17–24 million people worldwide and with a rising incidence each year (1, 2). CFS exerts a profound negative impact on patients’ quality of life and functional capacity. Approximately 35–69% are unable to maintain regular social participation or employment. Unfortunately, over 95% of CFS patients struggle to fully recover, and symptoms often persist lifelong (3). Consequently, CFS poses a multidimensional burden, substantially elevating economic and psychological distress for patients and their families while incurring considerable losses to broader societal productivity.

To date, no specific drug has been approved for CFS. Commonly used medications include acetylcholinesterase inhibitors, antidepressants, anti-inflammatory drugs, and immunomodulators (4, 5), but these are clearly not suitable for long-term symptom management and are accompanied by varying degrees of side effects (6, 7). Internationally, considerable research and hope have been invested in non-pharmacological therapies. Current management of CFS mainly focuses on symptoms. Cognitive-behavioral therapy (CBT) and graded exercise therapy (GET), which were previously recommended for CFS, are now considered to potentially exacerbate the condition (8, 9). The UK National Institute for Health and Care Excellence (NICE) guideline (10) suggests that GET should not be used, and psychological support (such as CBT) may help patients cope with symptoms, but not as a cure. In addition, graded exercise therapy (GET) are no longer advised the manner of fixed incremental increases. Both NICE (10) and recommendations from the US Centers for Disease Control and Prevention (CDC) (11) explicitly warn that unsupervised or excessive exercise may worsen CFS. In line with this, a recent Cochrane systematic review (12) concluded that exercise therapy probably has a modest effect in the short term and that long-term benefit or safety remains uncertain. These uncertainties highlight the need for exploring alternative non-pharmacological management strategies that are safe, sustainable, and tailored to the complex symptom profile of CFS.

Non-pharmacological Traditional Chinese Medicine (TCM) therapies, guided by holistic principles and meridian theory, have shown advantages in improving fatigue, sleep, and emotional symptoms, with safety, affordability, and minimal side effects (13, 14). In recent years, related clinical studies have proliferated, and previous meta-analyses suggest that acupuncture (15), moxibustion (16), tuina (massage) (17) may have potential therapeutic value for CFS. Although many non-pharmacological TCM therapies exist to relieve CFS-related symptoms, the optimal treatment is still debated, and current international guidelines do not specify which TCM-based interventions might be most effective for CFS. One previous network meta-analysis (15) compared the effectiveness of acupuncture therapies for CFS-related fatigue reduction, and another network meta-analysis (16) focused solely on depressive symptoms. These studies only focus on specific interventions or single symptom dimensions, making it challenging to provide comprehensive guidance for clinical practice. Therefore, a comprehensive overview of non-pharmacological TCM therapies is particularly important for patients with CFS.

Network Meta-analysis (NMA) enables indirect comparisons and quantitative analysis among interventions and can rank the efficacy of each therapy across multiple outcome measures, thereby providing valuable evidence for clinical decisions. The aim of this study was to compare and rank the therapeutic effects of different non-pharmacological TCM therapies in patients with CFS across multidimensional symptoms, clarify the symptom domains in which different interventions have advantages, and provide evidence for implementing individualized, stratified treatment strategies in clinical practice.

## 2 Methods

### 2.1 Standards and registration

This systematic review and NMA was reported according to the Preferred Reporting Items for Systematic reviews and Meta-Analysis guidelines (17), and this study was registered in PROSPERO (CRD420251113292).

### 2.2 Search strategy

Web of Science, Embase, PubMed, Scopus, CINAHL, Cochrane Library, CNKI, Wan Fang, VIP Database, and SinoMed were searched for eligible studies from inception to January 1, 2026. Additionally, the reference lists of included studies, as well as published systematic reviews and meta-analysis, were hand-searched to improve coverage (Supplementary material 2) outlines the details of the search strategy.

### 2.3 Inclusion and exclusion criteria

Inclusion and exclusion criteria were defined according to the PICOS framework (18). Articles meeting the following criteria were included: (1) parallel-group or cluster-randomized controlled trials involving adult patients (≥18 years old) with a precise diagnosis of CFS based on established criteria. Specifically, we accepted the 1994 CDC criteria (19), the Institute of Medicine (IOM) (20), the 2011 Introduction to International Consensus (ICC) (21), Holmes (22) or other explicitly stated diagnostic frameworks (23). A detailed breakdown of diagnostic criteria used by each included study is provided in Table 1. (2) The intervention group received non-pharmacological TCM therapies including acupuncture, moxibustion, massage, cupping, scraping, qigong, or acupoint application, as detailed in (Supplementary material 3). Control groups included either conventional interventions (usual care, no treatment/waiting list, non-specific symptomatic drugs, General advice) or inert treatment or active intervention controls (Different from the intervention group receiving non-pharmacological TCM therapies). (3) Assessment of at least one primary (overall fatigue) or secondary (physical fatigue, mental fatigue, sleep quality, anxiety, depression) outcome measure. Exclusion criteria were: (1) studies targeting specific populations (including but not limited to specific syndrome differentiation patterns, gender, or occupations). (2) Non-pharmacological interventions combined with drugs (oral medications, injectable drugs, or herbal medicines) or multi-drug combination interventions. (3) Studies that use modern medical interventions (e.g., cognitive behavioral therapy, graded exercise therapy, energy management therapy) as controls or interventions, or those comparing only different intensities or doses of the same intervention, were excluded to ensure intervention consistency and network robustness. (4) Studies classified as reviews, cross-over randomized controlled trials, secondary analysis, quasi-experimental studies, or conference abstracts; (5) Studies lacking full-text articles or insufficient data, even after contacting the corresponding author.

**Table 1:** Characteristics of included studies.

Study	Year	Region	Design	Diagnosis Criteria	Female (%)	Age (Year)	Intervention/Control	Sample size	Length of intervention (week)	Frequency	Outcome measures
Peng ZC	2024	China	Two-arm RCT	1994CDC	43.8	38.8 ± 16.5	Massage	48	4 week	12	FAI
					46.8	40.0 ± 18.3	Conventional care	47	4 week	84	
Lin YF	2021	China	Two-arm RCT	1994CDC	78.6	34 ± 9	Moxibustion	28	4 week	12	FS-14
					82.6	35 ± 9	Conventional care	29	NA	NA	
Li ZJ	2024	China	Two-arm RCT	1994CDC	58.06	31 ± 8	Massage	31	4 week	12	FS-14, PSQI
					68.57	32 ± 9	Conventional care	35	NA	NA	
Wang HN	2016	China	Two-arm RCT	1994CDC	48.7	36.49 ± 8.88	Massage	39	NA	10	FSS, PSQI
					50	37.32 ± 9.30	Conventional care	40	NA	10	
Wang JJ	2009	China	Two-arm RCT	1994CDC	59.4	35.8 ± 10.7	Acupuncture	32	4 week	12	FS-14
					59.4	38.8 ± 8.8	Inert treatment	32	4 week	12	
Liang H	2017	China	Two-arm RCT	1994CDC	63.33	41.16 ± 8.28	Acupuncture	30	4 week	20	FS-14, PSQI
					66.67	41.53 ± 8.64	Conventional care	30	4 week	12	
Chen XH	2010	China	Two-arm RCT	1994CDC	57.78	38.3 ± 9.9	Acupuncture	45	2 week	14	FS-14
					53.3	40.6 ± 9.5	Conventional care	45	2 week	14	
Ma J	2022	China	Two-arm RCT	1994CDC	31.48	39.48 ± 8.35	Moxibustion	54	4 week	12	FS-14, SDS
					35.19	39.52 ± 8.62	Conventional care	54	4 week	12	
J. S. Chan	2013	Hong Kong	Two-arm RCT	1994CDC	72.22	42.4 ± 6.7	Qigong	72	17 week	60	ChCFS, HADS
					81.53	42.5 ± 6.4	Conventional care	65	NA	NA	
Yu JY	2019	China	Two-arm RCT	1994CDC	46.67	45.2 ± 11.6	Cupping	30	4 week	14	FS-14, PSQI
					43.33	43.5 ± 12.7	Conventional care	30	4 week	28	
J. S. Chan	2014	Hong Kong	Two-arm RCT	1994CDC	61.3	39.1 ± 7.8	Qigong	75	9 week	16	ChCFS, PSQI, HADS
					82.7	38.9 ± 8.1	Conventional care	75	NA	NA	
Rainbow T H Ho	2012	Hong Kong	Two-arm RCT	1994CDC	75.8	42.1 ± 7.3	Qigong	33	17 week	60	ChCFS
					83.6	42.5 ± 5.5	Conventional care	31	NA	NA	
Li HN	2017	China	Two-arm RCT	1994CDC	43.59	41.8 ± 7.1	Massage	39	4 week	20	FS-14, HAMD, SAS
					36.84	42.63 ± 6.2	Acupuncture	38	4 week	20	
Ma S	2022	China	Two-arm RCT	1994CDC	53.3	37.23 ± 7.80	Acupuncture	30	4 week	24	FS-14, SDS, SAS
					50	37.70 ± 8.36	Conventional care	30	4 week	24	
Gong Y	2021	China	Two-arm RCT	1994CDC	63.3	42.85 ± 8.65	Scraping	30	4 week	5	FS-14, PSQI
					60	41.64 ± 7.88	Conventional care	30	4 week	30	
Shang K	2019	China	Two-arm RCT	1994CDC	51.4	31 ± 9.4	Massage	35	3 week	21	FAI
					54.3	30 ± 8.8	Conventional care	35	4 week	30	
Ma J	2018	China	Two-arm RCT	1994CDC	57.9	43 ± 6	Moxibustion	38	6 week	40	FAI
					47.4	43 ± 7	Acupuncture	38	6 week	40	
Hu Q	2016	China	Two-arm RCT	1994CDC	43.3	35.14 ± 3.51	Massage	30	6 week	20	FS-14, DSI, SAS
					50	36.14 ± 4.23	Acupuncture	30	6 week	20	
Tian L	2015	China	Two-arm RCT	1994CDC	33.3	42 ± 9	Moxibustion	36	4 week	30	FAI
					55.5	42 ± 10	Acupuncture	36	4 week	30	
Cheng XH	2014	China	Two-arm RCT	1994CDC	53.3	31.33 ± 5.94	Acupoint application	30	6 week	20	FS-14
					56.7	31.30 ± 5.68	Acupuncture	30	6 week	20	
Zhang W	2010	China	Two-arm RCT	1994CDC	45.5	48.0 ± 13.5	Acupuncture	22	4 week	20	ChCFS
					47.8	43.2 ± 12.4	Inert treatment	23	4 week	20	
Liu J	2022	China	Two-arm RCT	1994CDC	60	45.36 ± 2.13	Moxibustion	30	12 week	48	FS-14
					60.33	40.20 ± 12.67	Acupuncture	30	12 week	48	
Liang WL	2018	China	Two-arm RCT	1994CDC	60	40 ± 9	Moxibustion	35	4 week	24	FS-14
					65.7	41 ± 11	Conventional care	35	4 week	84	
Siu-Man Ng	2013	Hong Kong	Two-arm RCT	1994CDC	72	39.8 ± 6.6	Acupuncture	50	4 week	8	ChCFS
					65.3	42.0 ± 6.5	Inert treatment	49	4 week	8	
Chen XH	2011	China	Two-arm RCT	1994CDC	57.8	38 ± 10	Acupuncture	45	2 week	14	SAS
					53.3	41 ± 10	Conventional care	45	2 week	14	
Xu YX	2019	China	Two-arm RCT	1994CDC	73.53	39 ± 9	Acupuncture	34	4 week	20	FS-14, PSQI
					67.65	37 ± 9	Conventional care	34	4 week	20	
Xu YX	2018	China	Two-arm RCT	1994CDC	67.6	NA	Massage	37	4 week	12	FS-14, PSQI
					72.2	NA	Conventional care	36	4 week	12	
Dou FH	2023	China	Two-arm RCT	1994CDC	60	47.46 ± 1.93	Moxibustion	40	4 week	20	PSQI
					57.5	47.63 ± 1.99	Conventional care	40	4 week	28	
Doğukan Kurç	2025	Turkey	Two-arm RCT	1994CDC	70.6	26.64 ± 1.29	Massage	17	4 week	10	FSS, BDI
					72.7	29.54 ± 1.27	Conventional care	22	NA	NA	

CDC, Definition of the Center for Disease Control criteria; FAI, Fatigue Assessment Instrument; FS-14, 14-Item Fatigue Scale; HADS, Hospital Anxiety and Depression Scale; ChCFS, Chinese version of the Chalder Fatigue Scale; PSQI, Pittsburgh Sleep Quality Index; HAMD, Hamilton rating scale for depression; SAS, Self-rating Anxiety Scale; SDS, Self-rating Depression Scale; DSI, Depression Status Inventory; FSS, Fatigue Severity Scale; BDI, Beck Depression Inventory.

### 2.4 Study selection and data extraction

Four reviewers (Xu, Zi, Liu, and Guo) independently screened titles and abstracts. They retrieved full texts of potentially relevant studies and assessed their eligibility. Disagreements regarding study inclusion were resolved through discussion. The principal investigator (Zhang) double-checked the eligibility of the identified studies. The next step was full-text screening, completed by 4 investigators (Xu, Zi, Liu, and Guo), who independently reviewed the full texts of selected studies and assessed their eligibility based on the PICOS (population, intervention, comparison, outcomes, and study type) criteria.

### 2.5 Quality assessment

The Risk of bias was assessed using the RoB 2.0 tool across five domains (24): randomization, deviations from intended interventions, missing outcome data, outcome measurement, and selection of reported results. The overall risk of bias was classified as follows: (1) low risk of bias, if all domains were rated as low risk; (2) some concerns, if at least one domain raised some concerns but none was judged as high risk; and (3) high risk of bias, if at least one domain was judged as high risk, or if more than three domains were rated as having some concerns and this substantially reduced confidence in the results. Each study was independently assessed by two authors. Any discrepancies were resolved through collaborative discussion with a third author.

### 2.6 Statistical analysis

We conducted pairwise meta-analysis in Stata 18.0 to evaluate non-pharmacological TCM therapies vs. controls. Change-scores were analyzed with fixed or random-effects models, estimating effect sizes for fatigue, anxiety, and depression as SMDs with 95% CIs. The effect on sleep quality was estimated using WMD with 95% CIs. The degree of heterogeneity among the included studies was assessed using the I^2^ statistic; I^2^ = 0 indicated no heterogeneity. The low, medium, and high degrees of heterogeneity were defined as I^2^ values of 25%, 50, and 75%, respectively (25). If *p* > 0.1 and I^2^ < 50%, there is less heterogeneity, choose the fixed effects model for analysis. If I^2^ > 50%, there was obvious heterogeneity, and the random effect model was selected for combined analysis. We assessed this transitivity by examining mean age, percentage of female participants, baseline severity, illness duration, diagnostic criteria, sample size, outcome instrument, intervention dose/intensity, and comparator class across different treatment comparison groups (26). Assuming equal heterogeneity across all comparisons. Consistency refers to the agreement between direct and indirect evidence within the network. We assessed consistency using global methods (design-treatment inconsistency models) and local methods (SIDE) (27).

We conducted the network meta-analysis using Stata 18.0 within a frequentist framework with random-effects models, employing the netmeta and mvmeta packages. Final results generated a 2 × 2 comparison table and ranked interventions using the cumulative ranking curve (SUCRA) value. The SUCRA value reflects the relative ranking order among interventions and does not directly indicate the magnitude of effect size or the clinical significance of differences. A higher SUCRA value for an intervention indicates a greater likelihood of its superiority over others. However, whether this superiority is clinically meaningful depends on interpreting effect size estimates (SMD or MD) and their 95% confidence intervals. For outcomes with 10 or more studies, assess bias using the Egger test and a comparison-adjusted funnel plot to determine publication bias (28). To evaluate the robustness of our primary outcome, a sensitivity analysis was conducted. Studies with a sample size ≤ 30, an intervention duration < 4 weeks, and invasive interventions were excluded. Additionally, we assessed the quality of evidence for primary outcomes using the CINeMA (Confidence in Network Meta-Analysis) framework, which is based on the GRADE approach and considers six domains: study-internal bias, reporting bias, indirectness, imprecision, heterogeneity, and inconsistency. The CINeMA web application^1^ was used to perform the assessment and generate summary ratings. The credibility of the results was categorized into four levels: high, moderate, low, and very low (29).

## 3 Results

### 3.1 Search results

**Figure 1:**
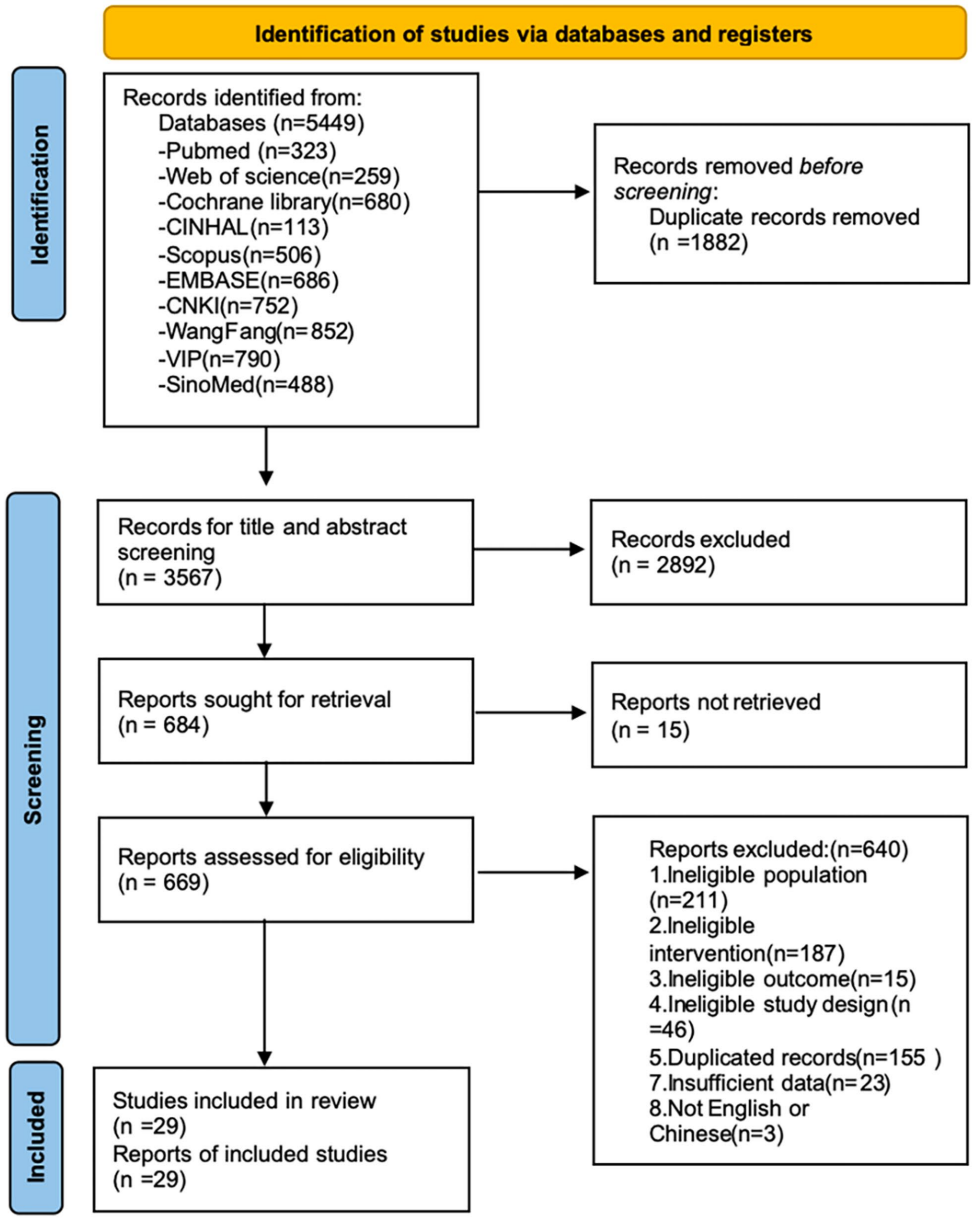
PRISMA flow diagram outlining the guideline selection process. CNKI, China national knowledge infrastructure; VIP, Chongqing VIP database.

Initially, 5,449 records were identified. After removing duplicates, 3,567 articles underwent title and abstract screening. Full-text reviews were conducted on 669 articles; ultimately, 29 studies involving 2,234 participants from 2009 to 2025 were included. The study selection process and flowchart of considered studies are shown in Figure 1.

### 3.2 Study characteristics

The main characteristics of the included studies are shown in Table 1. A total of 29 studies were included, comprising 7 English articles (18, 30–35), and 22 Chinese articles (36–57), encompassing 2,234 participants. Besides conventional cares (standard care, medication for fatigue relief, and no specific treatment for CFS) and inert treatment, seven different therapeutic approaches were evaluated. The average age of participants was 39.3 years, with comparable characteristics between the experimental and control groups. Regarding diagnostic criteria, all 29 included studies explicitly adopted the 1994 CDC criteria (19). No important concerns were raised regarding the violation of the transitivity assumption when the potential effect modifiers were evaluated. Characteristics of studies grouped by diagnostic criteria, illness duration, baseline severity, percentage of females, mean age, comparator class, sample size, intervention intensity, and outcome instrument across different treatment comparison groups are summarized in (Supplementary material 6).

### 3.3 Risk of bias assessment

The risk of bias assessment is summarized in (Supplementary material 4). Of the 29 included studies, 11 (37.9%) were rated as having some concerns, and 18 (62.1%) were rated as high risk of bias. The most common risks of bias included unclear randomization processes, unblinded assessors, and improper handling of missing data. No small-study effects were detected using the comparison-adjusted funnel plots and Egger test results (Supplementary material 8).

### 3.4 Pairwise meta-analysis

The pairwise meta-analysis for primary and secondary outcomes are presented in (Supplementary material 5). Compared with conventional care, moxibustion, massage, acupuncture, cupping, qigong, and scraping all improved overall fatigue (SMD = −2.91 to −0.38). Compared with conventional care, moxibustion, acupuncture, massage, and cupping effectively improved physical fatigue (SMD = −2.67 to −0.35). Similarly, compared with conventional care, massage, acupuncture, and qigong effectively reduced mental fatigue (SMD = −3.6 to −0.05). Cupping showed the best improvement in sleep quality (MD = −5.92 to −3.28) compared to conventional care. Regarding depression, acupuncture, moxibustion, qigong, and cupping all demonstrated better therapeutic effects than conventional cares (SMD = −1.76 to −0.12). Only moxibustion (SMD = −2.51 to −0.16) was observed to have a significant effect among the interventions for anxiety.

### 3.5 Consistency test

We performed global inconsistency and local consistency to identify inconsistency in the model. The results showed local consistency (*p* > 0.05) between direct and indirect comparisons for overall fatigue, mental fatigue, physical fatigue, sleep quality, anxiety, and depression. For the local inconsistency test, we use the node-splitting method to assess the local consistency, and all *p*-values from direct and indirect comparisons are greater than 0.05, indicating that the likelihood of local inconsistency is low. Finally, we use the consistency model for fitting. The tables are shown in (Supplementary material 7).

### 3.6 NMA for primary outcomes

#### 3.6.1 Overall fatigue

**Figure 2:**
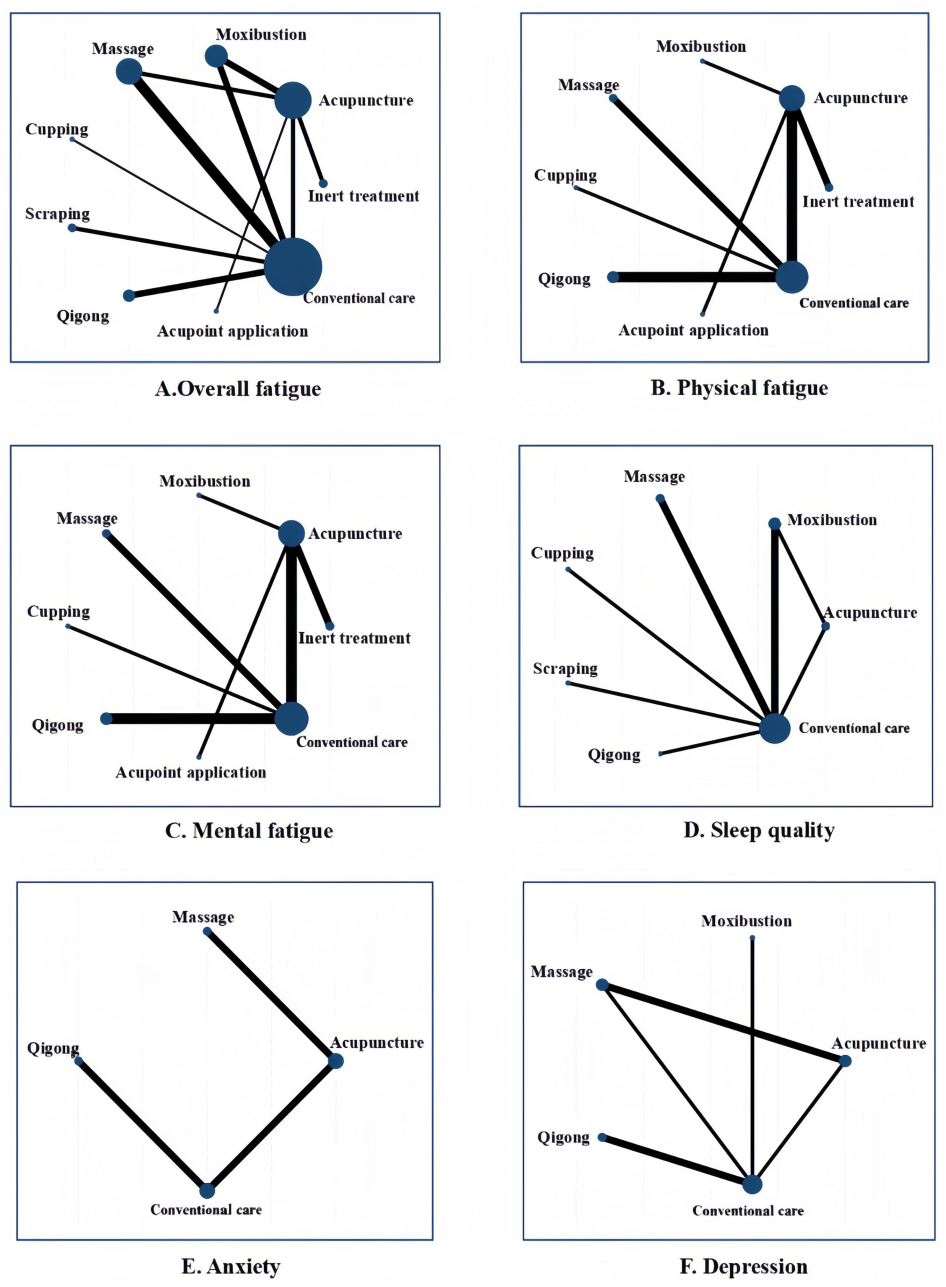
Network plot for all outcomes.

**Figure 3:**
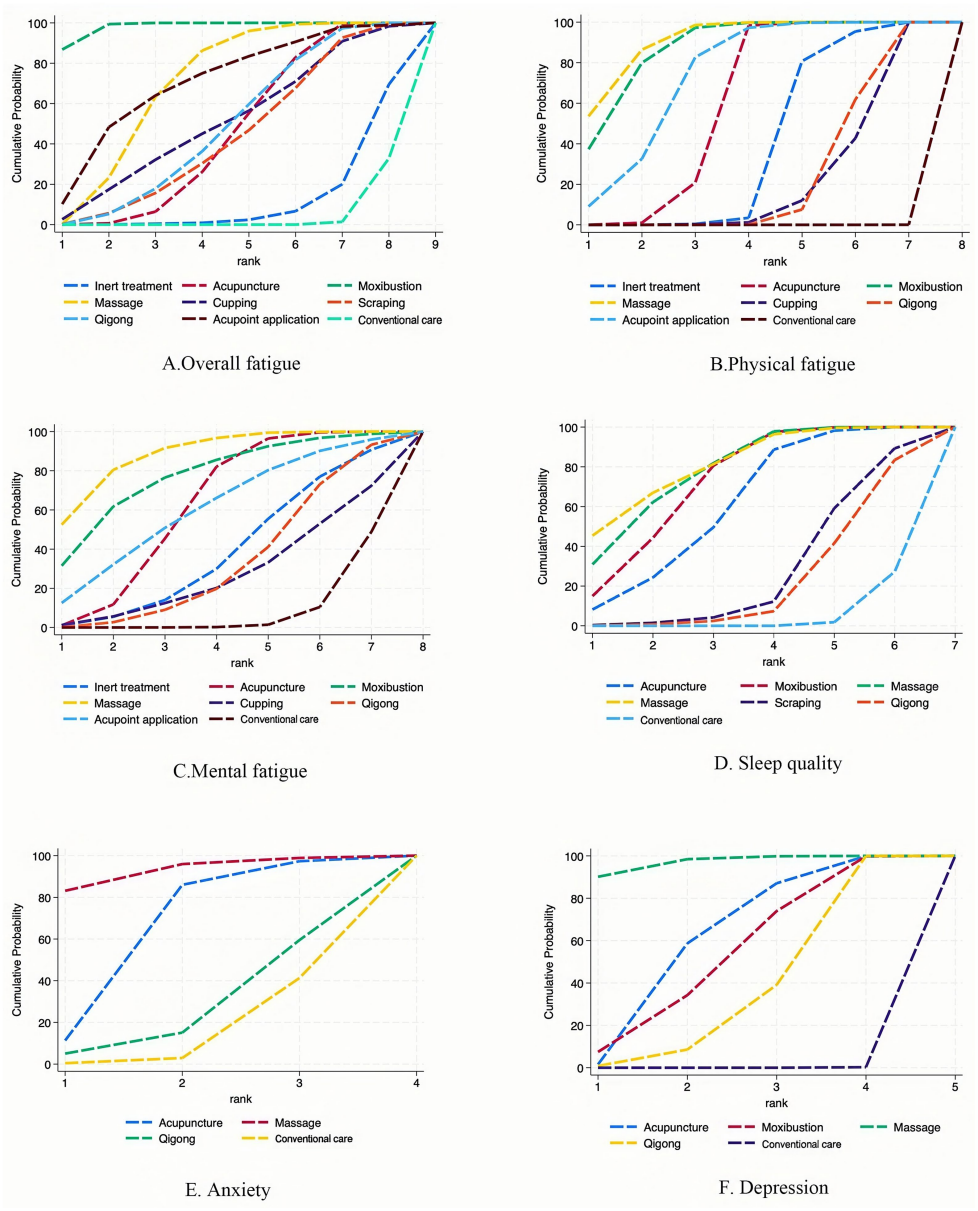
The efficacy cumulative rank probabilities for all outcomes.

Figure 2A presents the network plot for overall fatigue. A total of 24 studies with 1,789 patients reported outcomes related to overall fatigue, associated with seven non-pharmacological TCM therapies: acupuncture, moxibustion, massage, cupping, scraping, qigong, and acupoint application. The comparison-adjusted funnel plot of overall fatigue and the Egger test indicated no bias (Supplementary material 8). We further conducted a network meta-analysis of the overall fatigue across included studies and generated a ranking table of evidence (Table 2), revealing 14 statistically significant pairwise comparisons. The cumulative ranking probability plot for overall fatigue (Figure 3A) showed SUCRA probability values, including moxibustion (98.3%), acupoint application (71.0%), massage (70.6%), cupping (51.4%), acupuncture (46.5%), qigong (49.5%), and scraping (44.9%).

**Table 2:** League table for overall fatigue and physical fatigue.

	Physical fatigue								
Overalll fatigue	**Inert treatment**	**−0.36** **(−0.68,−0.04)**	**−0.86** **(−1.47,−0.24)**	**−0.94** **(−1.57,−0.30)**	0.40 (−0.30,1.11)	-	0.34 (−0.16,0.85)	−0.60 (−1.21,0.01)	**1.27** **(0.82,1.73)**
	**0.65** **(0.03,1.28)**	**Acupuncture**	−0.50 (−1.02,0.03)	**−0.58** **(−1.12,−0.03)**	**0.77 (0.14,1.39)**	-	0.71 (0.30,1.11)	−0.24 (−0.76,0.28)	**1.63** **(1.32,1.95)**
	**1.66** **(0.91,2.40)**	**1.00** **(0.60,1.40)**	**Moxibustion**	−0.08 (−0.83,0.68)	**1.26 (0.44,2.08)**	-	**1.20 (0.54,1.87)**	0.26 (−0.48,1.00)	**2.13** **(1.52,2.74)**
	**0.95** **(0.20,1.70)**	0.30 (−0.12,0.72)	**−0.71** **(−1.19,−0.22)**	**Massage**	**1.34 (0.64,2.04)**	-	**1.28 (0.76,1.80)**	0.34 (−0.41,1.09)	**2.21** **(1.77,2.65)**
	0.71 (−0.42,1.84)	0.06 (−0.89,1.00)	−0.95 (−1.90,0.01)	−0.24 (−1.17,0.69)	**Cupping**	-	−0.06 (−0.66,0.54)	**−1.00** **(−1.82,−0.19)**	**0.87** **(0.33,1.41)**
	0.62 (−0.34,1.57)	−0.04 (−0.76,0.68)	**−1.04** **(−1.77,−0.31)**	−0.33 (−1.03,0.37)	−0.09 (−1.15,0.97)	**Scraping**	-	-	-
	0.68 (−0.18,1.55)	0.03 (−0.57,0.63)	**−0.97** **(−1.58,−0.36)**	−0.26 (−0.84,0.31)	−0.02 (−1.01,0.96)	0.07 (−0.69,0.83)	**Qigong**	**−0.94** **(−1.60,−0.28)**	**0.93** **(0.66,1.20)**
	1.04 (−0.02,2.10)	0.39 (−0.46,1.24)	−0.61 (−1.56,0.33)	0.09 (−0.86,1.04)	0.33 (−0.94,1.61)	0.43 (−0.69,1.54)	0.36 (−0.68,1.40)	**Acupoint application**	**1.87** **(1.26,2.48)**
	−0.19 (−0.92,0.54)	**−0.84** **(−1.22,−0.46)**	**−1.84** **(−2.25,−1.44)**	**−1.14** **(−1.48,−0.80)**	**−0.90 (** **−1.76,−0.03)**	**−0.80** **(−1.41,−0.19)**	**−0.87** **(−1.33,−0.41)**	**−1.23** **(−2.16,−0.29)**	**Conventional care**

Green color means treatment; flesh color means the league table for overall fatigue; blue color means the league table for Physical fatigue.

### 3.7 NMA for secondary outcomes

#### 3.7.1 Physical fatigue

Figure 2B shows the network plot for physical fatigue. 13 studies with 1,051 patients reported outcomes related to physical fatigue in association with six non-pharmacological TCM therapies: acupuncture, moxibustion, massage, cupping, qigong, and acupoint application. The comparison-adjusted funnel plot of the physical fatigue and the Egger test indicated no bias (Supplementary material 8). We further conducted a network meta-analysis of physical fatigue across the included studies generated a ranking table of evidence (Table 2), which revealed 18 statistically significant pairwise comparisons. The cumulative ranking probability plot for physical fatigue (Figure 3B) showed SUCRA probability values, including massage (91.2%), moxibustion (87.5%), acupoint application (74.3%), acupuncture (60.2%), qigong (24.0%), and cupping (22.3%).

#### 3.7.2 Mental fatigue

Figure 2C presents the network plot for mental fatigue. 13 studies with 1,051 patients reported outcomes related to mental fatigue and six non-pharmacological TCM therapies: acupuncture, moxibustion, massage, cupping, qigong, and acupoint application. The comparison-adjusted funnel plot of mental fatigue and the Egger test showed no bias (Supplementary material 8). We further conducted a network meta-analysis of mental fatigue across included studies and generated a ranking table for mental fatigue evidence outcomes (Table 3), revealing 4 statistically significant pairwise comparisons. The cumulative ranking probability plot for mental fatigue (Figure 3C) showed SUCRA probability values, including massage (88.7%), moxibustion (78.1%), acupuncture (62.5%), acupoint application (61.3%), qigong (34.4%), and cupping (27.7%).

**Table 3:** League table for mental fatigue and sleep quality.

	Sleep quality								
Mental fatigue	**Inert treatment**	-	-	-	-	-	-	-	-
	0.50 (−0.48,1.48)	**Acupuncture**	−0.47 (−2.32,1.39)	−0.80 (−3.38,1.77)	−1.02 (−4.09,2.06)	2.11 (−0.92,5.15)	2.48 (−0.53,5.50)	-	**3.58** **(1.73,5.44)**
	1.02 (−0.69,2.74)	0.52 (−0.88,1.93)	**Moxibustion**	−0.33 (−2.65,1.99)	−0.55 (−3.41,2.32)	2.58 (−0.24,5.40)	**2.95 (0.15,5.75)**	-	**4.05** **(2.57,5.53)**
	1.34 (−0.30,2.97)	0.84 (−0.47,2.15)	0.31 (−1.61,2.23)	**Massage**	−0.22 (−3.25,2.82)	2.91 (−0.07,5.90)	**3.28 (0.32,6.25)**	-	**4.38** **(2.60,6.16)**
	−0.36 (−2.26,1.54)	−0.86 (−2.48,0.77)	−1.38 (−3.53,0.77)	−1.70 (−3.43,0.04)	**Cupping**	3.13 (−0.30,6.56)	**3.50 (0.09,6.91)**	-	**4.60** **(2.15,7.05)**
	-	-	-	-	-	**Scraping**	0.37 (−3.00,3.74)	-	1.47 (−0.93,3.87)
	−0.14 (−1.64,1.36)	−0.64 (−1.78,0.49)	−1.17 (−2.98,0.64)	**−1.48** **(−2.77,−0.19)**	0.22 (−1.40,1.83)	-	**Qigong**	-	1.10 (−1.27,3.47)
	0.56 (−1.15,2.27)	0.06 (−1.34,1.47)	−0.46 (−2.45,1.53)	−0.77 (−2.69,1.15)	0.92 (−1.23,3.07)	-	0.71 (−1.10,2.51)	**Acupoint application**	-
	−0.71 (−1.99,0.56)	**−1.21** **(−2.02,−0.39)**	**−1.73** **(−3.36,−0.11)**	**−2.05** **(−3.07,−1.02)**	−0.35 (−1.76,1.06)	-	−0.57 (−1.36,0.22)	−1.27 (−2.90,0.35)	**Conventional care**

Green color means treatment; flesh color means the league table for Mental fatigue; blue color means the league table for Sleep quality.

#### 3.7.3 Sleep quality

Figure 2D presents the network plot for sleep quality. A total of 9 studies with 687 patients reported sleep quality outcomes for 6 non-pharmacological TCM therapies: acupuncture, moxibustion, massage, cupping, scraping, and qigong. We also performed a network meta-analysis of sleep quality across included studies and generated a ranking table for sleep quality outcomes (Table 3), revealing 7 statistically significant pairwise comparisons. The cumulative ranking probability plot for sleep quality (Figure 3D) showed SUCRA probability values, including cupping (81.2%), massage (79.3%), moxibustion (73.2%), acupuncture (61.8%), scraping (28.0%), and qigong (22.0%).

#### 3.7.4 Anxiety

Figure 2E displays the network plot of anxiety. A total of 6 studies involving 574 patients reported anxiety outcomes related to three non-pharmacological TCM therapies: acupuncture, massage, and qigong. We also conducted a network meta-analysis of anxiety across the included studies and generated a ranking table for anxiety outcome evidence. (Table 4). Only the comparison of massage vs. conventional care demonstrated statistical significance. The cumulative ranking probability plot for anxiety (Figure 3E) showed SUCRA values including: massage (92.0%), acupuncture (64.9%), and qigong (26.1%).

**Table 4:** League table for anxiety and depression.

	Anxiety				
Depression	**Acupuncture**	-	−1.33 (−3.65,0.99)	1.75 (−1.53,5.03)	2.02 (−0.32,4.36)
	−0.12 (−0.81,0.58)	**Moxibustion**	-	-	-
	**0.38** **(0.01,0.75)**	0.50 (−0.22,1.22)	**Massage**	3.08 (−0.94,7.09)	**3.35** **(0.05,6.64)**
	−0.28 (−0.87,0.31)	−0.16 (−0.73,0.41)	**−0.66** **(−1.28,−0.04)**	**Qigong**	0.27 (−2.03, 2.57)
	**−0.85** **(−1.35,−0.34)**	**−0.73** **(−1.21,−0.25)**	**−1.23** **(−1.76,−0.69)**	**−0.57** **(−0.88,−0.26)**	**Conventional care**

Change-scores were used in each league table. Data for overall fatigue, physical fatigue mental fatigue, anxiety, depression are reported as SMD, with 95% CI, Data for sleep quality are reported as MD, with 95% CI. Each value in the cells represents the effect size between two treatments. Higher values indicate worse symptom severity; therefore, negative effect estimates favor the intervention listed in the column compared with the row intervention. Statistically significant differences are highlighted in bold (*p* < 0.05). Estimates are derived from network meta-analysis combining direct and indirect evidence. Green color means treatment; flesh color means the league table for Depression; blue color means the league table for Anxiety.

#### 3.7.5 Depression

Figure 2F shows the network plot of depression. A total of 7 studies involving 631 patients reported outcomes related to depression for 4 non-pharmacological TCM therapies: acupuncture, moxibustion, massage, and qigong. We further conducted a network meta-analysis of depression across the included studies. We generated a ranking table for depression evidence outcomes (Table 4), where 6 pairwise comparisons were statistically significant. The cumulative ranking probability plot for depression (Figure 3F) showed probability SUCRA values, including massage (97.0%), acupuncture (62.1%), moxibustion (53.9%), and qigong (36.9%).

### 3.8 Additional analysis

The sensitivity analysis based on the sample size, intervention method, and intervention duration for overall fatigue was generally consistent with the original analysis (Supplementary material 12). However, the certainty of evidence for these interventions in improving overall fatigue was very low to moderate. Evidence was downgraded mainly due to concerns about within-study bias, with major concerns identified in 70.8% of comparisons. Heterogeneity was also a common reason for downgrading, with 29.2% of comparisons showing some concerns. Overall, the evidence was minimally affected by indirectness and incoherence. The table are shown in Supplementary material 13.

## 4 Discussion

This systematic review and NMA focus on evaluating the comparative effectiveness of non-pharmacological TCM therapies compared with conventional care for alleviating CFS symptoms in adults and should therefore be interpreted as evidence supporting treatment selection within the non-pharmacological TCM clinical framework. The results indicate that seven non-pharmacological TCM therapies, encompassing acupuncture, moxibustion, massage, cupping, scraping, qigong, and acupoint application, demonstrate differential clinical efficacy against the diverse symptoms experienced by patients with CFS. For overall fatigue, moxibustion ranked highest (SUCRA = 98.3%), indicating a higher likelihood of being among the more effective interventions. For physical fatigue (SUCRA = 91.1%), mental fatigue (SUCRA = 89.0%), anxiety (SUCRA = 92.0%), and depression (SUCRA = 97.0%), massage ranked relatively high compared with other interventions. Additionally, for sleep quality, cupping ranked highest (SUCRA = 81.3%). Overall, moxibustion showed a higher probability of improving overall fatigue, while massage ranked higher across several fatigue-related and psychological outcomes. Cupping ranked highest for sleep quality within the current network. However, these rankings should be interpreted cautiously and considered alongside the effect estimates and their associated uncertainty.

This study found that moxibustion was associated with greater improvements in overall fatigue compared to conventional care, consistent with a previous meta-analysis (58). In TCM theory, CFS is called ‘Xu Lao’ (Consumptive Disease), a condition characterized by a deficiency of vital energy (Qi). Moxibustion is traditionally believed to warm Yang and tonify deficiency, which aligns with therapeutic principles for fatigue-related disorders (15). From a modern biomedical perspective, some experimental studies have suggested that moxibustion may influence multiple physiological pathways, including immune regulation, mitochondrial energy metabolism (59), and neuroendocrine activity. For example, animal studies have reported that moxibustion may regulate hypothalamic–pituitary–adrenal (HPA) axis activity and modulate immune markers such as IgA, IgG, and complement C3 (60). Another study has suggested that moxibustion may activate signaling pathways related to mitochondrial energy metabolism, such as AMPK/PGC-1α (61). These findings indicate that moxibustion may potentially influence biological processes, thereby reducing overall fatigue (62). A recent clinical research study (63) indicates that moxibustion may improve fatigue in CFS patients by modulating the vagus nerve, with its long-term effects surpassing those of acupuncture. These effects align well with moxibustion’s ranking in overall fatigue management.

Notably, this meta-analysis suggests that among various non-pharmacological TCM therapies, massage therapy may be a more effective overall treatment for improving the four outcomes in CFS patients: mental fatigue, physical fatigue, anxiety, and depression. While moxibustion targets deep energy production issues and functional suppression in CFS, massage could have broader effects on multiple symptom domains within the current treatment network. The four symptoms of CFS are interconnected and may form a closely linked symptom cluster network (64–67). Physical and mental fatigue are physiological burdens on emotions, while persistent anxiety and depression can exacerbate energy depletion and cognitive fatigue through neuroendocrine mechanisms, creating a vicious cycle (68–70). Therefore, a therapy that can simultaneously improve multidimensional symptoms may be better aligned with the complex pathology of CFS.

Although the present network meta-analysis cannot directly determine the biological mechanisms underlying these effects, several hypothetical explanations have been proposed in previous experimental and clinical studies. First, evidence from physiological studies indicates that massage can modulate HPA axis activity and autonomic balance. A study (71) demonstrated that massage significantly reduced serum cortisol and norepinephrine levels while increasing heart rate variability (HRV), suggesting suppressed sympathetic nervous system activity and enhanced vagal tone. Field et al. (72) further reported that massage typically decreases cortisol levels by approximately 31%, with increases in serotonin and dopamine of about 28 and 31%, respectively. These neuroendocrine changes are closely associated with improved mood, reduced stress perception, and reduced fatigue. Mechanistically, massage provides continuous mechanical stimulation of cutaneous and muscular mechanoreceptors, activating spinal–brainstem pathways that influence central autonomic regulation. Through this process, massage is thought to normalize HPA axis reactivity and rebalance sympathetic–parasympathetic activity, promoting the release of “comfort-related” neurotransmitters such as endorphins and serotonin while inhibiting excessive stress hormone secretion. These effects may directly contribute to improvements in mental well-being, sleep quality, and perceived energy levels. Second, massage may alleviate fatigue and psychological symptoms by reducing inflammatory responses. Evidence suggests that fatigue and affective symptoms may share common biological substrates, particularly the activation of pro-inflammatory cytokines networks. Experimental studies (69) have shown that the administration or induction of pro-inflammatory cytokines is associated with increased depressive mood, fatigue, and sleep disturbances. Massage might help decrease the release of inflammatory mediators, including cytokines, thereby mitigating inflammation-related fatigue and emotional symptoms. In addition to neuroendocrine and immunological regulation, massage enhances local tissue microcirculation, facilitating the clearance of fatigue-related metabolites and further contributing to symptom relief (73). A recent RCT study (74) and previous systematic review (75, 76) also consistently support the efficacy of massage therapy for fatigue and emotional disorders. A meta analysis (77) indicated that Massage can effectively reduce trait anxiety and depression, and the therapeutic effect of one course of treatment is comparable to that of psychotherapy. Compared to acupuncture, which mainly relies on acupoint specificity, massage may provide continuous, gentle mechanical stimulation and tactile emotional connection on the body’s surface. This may allow it to systematically reduce physical and mental fatigue and relieve anxiety and depression by enhancing autonomic nerve function, regulating hormone levels, and releasing positive emotion neurotransmitters. Our findings suggest that for CFS subtypes or symptom groups characterized by fatigue and emotional comorbidity, massage could be an effective primary intervention that triggers a cascade effect. Future research should conduct carefully designed mechanistic experiments to further verify the specific effects of massage on HPA axis function, inflammatory factor profiles, and brain functional network connectivity in CFS patients, to confirm its systemic regulatory role.

Cupping therapy ranked highest in the probability ranking for improving sleep quality among the included interventions, similar to the findings of a previous study (78). Several mechanisms have been proposed to explain the potential effects of cupping on sleep and relaxation. Cupping therapy promotes local blood vessel dilation and blood circulation through negative pressure, stimulates the metabolism of the skin and superficial tissues, and relieves muscle tension and pain, thereby producing a relaxation effect and helping patients fall asleep (79). It may also enhance the metabolism of substances that cause pain, thereby reducing muscle tension and discomfort and creating a favorable environment for sleep. Neuroimaging studies (80) have further suggested that cupping may alter functional connectivity in brain regions involved in emotional and sleep regulation, such as the hippocampus and medial prefrontal cortex. Interestingly, cupping therapy did not rank among the top performers for other outcomes. In particular, for physical and mental fatigue, its effect was even worse than inert treatment. This discrepancy may stem from the varying degrees of matching between the underlying pathology of different symptoms and the specific mechanisms of the intervention. Cupping is a high-intensity sensory stimulation technique. Unlike moxibustion, it does not directly restore energy, nor does it offer continuous gentle mechanical stimulation and tactile emotional interactions like massage. Instead, it may rapidly disrupt the pain-tension-insomnia cycle through a reset or interference mechanism and stimulate the neural-endocrine-immune network by modulating local immune responses (81), thus showing effectiveness in improving sleep quality. Chronic fatigue involves significant physical and mental energy depletion. Cupping therapy may help conserve energy indirectly by improving blood flow, immune response, and pain relief, but it cannot directly restore energy or repair functions. However, the existing evidence remains limited, and the extent to which these mechanisms contribute to improvements in sleep quality in CFS remains unclear.

Importantly, the potential mechanisms described above were not directly evaluated in the randomized trials included in this network meta-analysis. Therefore, these interpretations should be considered exploratory rather than definitive explanations of the observed treatment rankings. Further mechanistic and clinical studies are required to clarify the roles of different therapies in managing symptoms in patients with CFS in the future.

This NMA is subject to certain limitations that should be acknowledged. First, heterogeneity is a significant limitation of this analysis. Moderate to substantial heterogeneity was observed in pairwise comparisons across several outcomes, potentially due to the difficulty of implementing participant blinding in non-pharmacological interventions. For subjective outcomes such as fatigue, anxiety, and sleep quality, lack of blinding may lead to expectation-related effects and potentially inflate estimated treatment benefits. In addition, most included trials were relatively small randomized studies conducted in China, which may introduce small-study effects and limit the generalizability of the findings. Therefore, the pooled effects and SUCRA rankings should be interpreted cautiously. Second, the risk of bias in the included studies was graded as some concerns or high, which reduced the certainty of evidence. Therefore, future research is likely to change the existing effect sizes and treatment rankings (29). Third, although treatments were classified based on prior systematic reviews or other NMAs (82–84), combining routine care, no treatment/waiting lists, non-specific symptomatic drugs and general advice (such as education, reassurance, exercise, and bed rest) (85) into a single summary node may lead to heterogeneity. Thus, the SUCRA rankings shown in this study represent probabilistic treatment rankings within the network and should not be viewed as definitive evidence of clinical superiority. Fourth, the study’s network was limited to non-pharmacological TCM therapies and did not compare these with other guideline-based non-pharmacological treatments, such as cognitive behavioral therapy or energy management strategies. Therefore, caution should be taken when interpreting the findings within the broader context of overall CFS treatment. Fifth, sensitivity analyses were only conducted for the overall fatigue outcome because it included the largest number of eligible studies. For other outcomes, the limited number of trials led to sparse treatment networks, which may compromise the stability of the sensitivity analyses. Future studies with larger sample sizes and more comprehensive outcome reporting are necessary to further validate these findings.

## 5 Conclusion

This network meta-analysis provides a quantitative synthesis of the available evidence on non-pharmacological TCM therapies for adults with chronic fatigue syndrome. Within the TCM-based treatment network included in this study, moxibustion was associated with greater improvements in overall fatigue compared with conventional care. Massage ranked highest for physical fatigue, mental fatigue, anxiety, and depression, while cupping ranked highest for sleep quality in the probability ranking. These findings suggest that different non-pharmacological TCM therapies may have varying effects across symptom domains. However, the results should be interpreted cautiously given the uncertainty of the available evidence and the limitations of the included studies. Further large-scale, high-quality randomized controlled trials are needed to confirm these findings and to clarify the role of these therapies within the broader management of CFS.

## Data Availability

The original contributions presented in the study are included in the article/Supplementary material, further inquiries can be directed to the corresponding authors.

## Footnotes

1
https://cinema.ispm.unibe.ch/
