# Supplementary material for: Comparative effectiveness of non-pharmacological traditional Chinese medicine therapies for chronic fatigue syndrome: a systematic review and network meta-analysis
Source: Front Med (Lausanne). 2026 Apr 1;13:1804710. doi: 10.3389/fmed.2026.1804710 (PMC13079024; doi:10.3389/fmed.2026.1804710)
Supplement: Supplementary file 1 [file Supplementary_file_1.docx]

Supplementary Material

**Effectiveness of Non-pharmacological Traditional Chinese Medicine Therapies for** **Chronic Fatigue Syndrome****: A Systematic Review and Network Meta-Analysis**

Yihan Zhang^1^, You Zhou^2^, Hangying Xu^1^, Chenxin Zhang^1^, Lingyi Guo^1^, Aiqun Zi^1^, Yan Xu^2^, Min Xu^2*^, Ting Liu^1*^

**Index of supplementary materials**

Supplementary 1. PRISMA checklist

Supplementary 2. Search strategy

Supplementary 3. Additional details on Methods

Supplementary 4. Risk of bias assessment for the included studies

Supplementary 5. Pairwise meta-analyses

5.1. Pairwise meta-analyses: Overall Fatigue

5.2. Pairwise meta-analyses: Physical Fatigue

5.3. Pairwise meta-analyses: Mental Fatigue

5.4. Pairwise meta-analyses: Sleep Quality

5.5. Pairwise meta-analyses: Anxiety

5.6. Pairwise meta-analyses: Depression

Supplementary 6. Assessment of transitivity

Supplementary 7. Assessment of inconsistency

7.1. Estimated global inconsistency in networks

7.2. Estimated local inconsistency in networks

Supplementary 8. Comparison-adjusted funnel plots

Figure S8.1. The funnel plot for Overall Fatigue

Figure S8.2. The funnel plot for Physical Fatigue

Figure S8.3. The funnel plot for Mental Fatigue

Supplementary 9. Network plots

9.1. The network plot for Overall Fatigue

9.2. The network plot for Physical Fatigue

9.3. The network plot for Mental Fatigue

9.4. The network plot for Anxiety

9.5. The network plot for Depression

Supplementary 10. Rank results and SUCRA

10.1. Rank results and SUCRA for Overall Fatigue

10.2. Rank results and SUCRA for Physical Fatigue

10.3. Rank results and SUCRA for Mental Fatigue

10.4. Rank results and SUCRA for Sleep quality

10.5. Rank results and SUCRA for Anxiety

10.6. Rank results and SUCRA for Depression

Supplementary 11. League tables of the network meta-analysis

11.1. The league table for Overall Fatigue

11.2. The league table for Physical Fatigue

11.3. The league table for Mental Fatigue

11.4. The league table for Sleep quality

11.5. The league table for Anxiety

11.6. The league table for Depression

Supplementary 12. Sensitivity analyses for Overall Fatigue

Table S12.a. Overall Fatigue removing studies with Invasive intervention

Table S12.b. Overall Fatigue studies removing studies with a sample size of≤30

Table S12.c. Overall Fatigue studies removing studies with studies with an intervention duration ≤4 weeks

Supplementary 13. Grading the evidence using CINeMA for Fatigue

Table S13.1. CINEMA Assessments for Overall Fatigue

Table S13.2. CINEMA Assessments for Physical Fatigue

Table S13.3. CINEMA Assessments for Mental Fatigue

**Supplementary 1. PRISMA checklist**

| **Section/Topic** | **Item #** | **Checklist Item** | **Reported on Page #** |
| --- | --- | --- | --- |
| **TITLE** |  |  |  |
| Title | 1 | Identify the report as a systematic review *incorporating a network meta-analysis (or related form of meta-analysis).* | **1** |
|  |  |  |  |
| **ABSTRACT** |  |  |  |
| Structured summary | 2 | Provide a structured summary including, as applicable:  **Background:** main objectives  **Methods:** data sources; study eligibility criteria, participants, and interventions; study appraisal; and *synthesis methods, such as network meta-analysis.*  **Results:** number of studies and participants identified; summary estimates with corresponding confidence/credible intervals; *treatment rankings may also be discussed. Authors may choose to summarize pairwise comparisons against a chosen treatment included in their analyses for brevity.*  **Discussion/Conclusions:** limitations; conclusions and implications of findings.  **Other:** primary source of funding; systematic review registration number with registry name. | **1** |
|  |  |  |  |
| **INTRODUCTION** |  |  |  |
| Rationale | 3 | Describe the rationale for the review in the context of what is already known*, including mention of why a network meta-analysis has been conducted.* | **2** |
| Objectives | 4 | Provide an explicit statement of questions being addressed, with reference to participants, interventions, comparisons, outcomes, and study design (PICOS). | **2** |
|  |  |  |  |
| **METHODS** |  |  |  |
| Protocol and registration | 5 | Indicate whether a review protocol exists and if and where it can be accessed (e.g., Web address); and, if available, provide registration information, including registration number. | **2** |
| Eligibility criteria | 6 | Specify study characteristics (e.g., PICOS, length of follow-up) and report characteristics (e.g., years considered, language, publication status) used as criteria for eligibility, giving rationale. *Clearly describe eligible treatments included in the treatment network, and note whether any have been clustered or merged into the same node (with justification).* | **Supplementary 3;**  **2, 5** |
| Information sources | 7 | Describe all information sources (e.g., databases with dates of coverage, contact with study authors to identify additional studies) in the search and date last searched. | **5** |
| Search | 8 | Present full electronic search strategy for at least one database, including any limits used, such that it could be repeated. | **Supplementary 2;** |
| Study selection | 9 | State the process for selecting studies (i.e., screening, eligibility, included in systematic review, and, if applicable, included in the meta-analysis). | **5** |
| Data collection process | 10 | Describe method of data extraction from reports (e.g., piloted forms, independently, in duplicate) and any processes for obtaining and confirming data from investigators. | **5** |
| Data items | 11 | List and define all variables for which data were sought (e.g., PICOS, funding sources) and any assumptions and simplifications made. | **5** |
| **Geometry of the network** | **S1** | Describe methods used to explore the geometry of the treatment network under study and potential biases related to it. This should include how the evidence base has been graphically summarized for presentation, and what characteristics were compiled and used to describe the evidence base to readers. | **Supplementary3;**  **5** |
| Risk of bias within individual studies | 12 | Describe methods used for assessing risk of bias of individual studies (including specification of whether this was done at the study or outcome level), and how this information is to be used in any data synthesis. | **Supplementary 4**  **5** |
| Summary measures | 13 | State the principal summary measures (e.g., risk ratio, difference in means). *Also describe the use of additional summary measures assessed, such as treatment rankings and surface under the cumulative ranking curve (SUCRA) values, as well as modified approaches used to present summary findings from meta-analyses.* | **5** |
| Planned methods of analysis | 14 | Describe the methods of handling data and combining results of studies for each network meta-analysis. This should include, but not be limited to:   - *Handling of multi-arm trials;* - *Selection of variance structure;* - *Selection of prior distributions in Bayesian analyses; and* - *Assessment of model fit.* | **5** |
| **Assessment of Inconsistency** | **S2** | Describe the statistical methods used to evaluate the agreement of direct and indirect evidence in the treatment network(s) studied. Describe efforts taken to address its presence when found. | **5** |
| Risk of bias across studies | 15 | Specify any assessment of risk of bias that may affect the cumulative evidence (e.g., publication bias, selective reporting within studies). | **5** |
| Additional analyses | 16 | Describe methods of additional analyses if done, indicating which were pre-specified. This may include, but not be limited to, the following:   - Sensitivity or subgroup analyses; - Meta-regression analyses; - *Alternative formulations of the treatment network; and* - *Use of alternative prior distributions for Bayesian analyses (if applicable).* | **5** |
|  |  |  |  |
| **RESULTS†** |  |  |  |
| Study selection | 17 | Give numbers of studies screened, assessed for eligibility, and included in the review, with reasons for exclusions at each stage, ideally with a flow diagram. | **Figure 1;5** |
| **Presentation of network structure** | **S3** | Provide a network graph of the included studies to enable visualization of the geometry of the treatment network. | **Figure 3;**  **Supplementary 9** |
| **Summary of network geometry** | **S4** | Provide a brief overview of characteristics of the treatment network. This may include commentary on the abundance of trials and randomized patients for the different interventions and pairwise comparisons in the network, gaps of evidence in the treatment network, and potential biases reflected by the network structure. | **5**  **Supplementary6;** |
| Study characteristics | 18 | For each study, present characteristics for which data were extracted (e.g., study size, PICOS, follow-up period) and provide the citations. | **Table 1;**  **3,4** |
| Risk of bias within studies | 19 | Present data on risk of bias of each study and, if available, any outcome level assessment. | **Supplementary 4;**  **6** |
| Results of individual studies | 20 | For all outcomes considered (benefits or harms), present, for each study: 1) simple summary data for each intervention group, and 2) effect estimates and confidence intervals. *Modified approaches may be needed to deal with information from larger networks.* | **Supplementary 5;**  **6** |
| Synthesis of results | 21 | Present results of each meta-analysis done, including confidence/credible intervals. *In larger networks, authors may focus on comparisons versus a particular comparator (e.g. placebo or standard care), with full findings presented in an appendix. League tables and forest plots may be considered to summarize pairwise comparisons.* If additional summary measures were explored (such as treatment rankings), these should also be presented. | **Supplementary 10;**  **Supplementary 11;**  **Table 2;**  **Table 3;**  **Table 4**  **Figure 4;**  **7-11** |
| **Exploration for inconsistency** | **S5** | Describe results from investigations of inconsistency. This may include such information as measures of model fit to compare consistency and inconsistency models, *P* values from statistical tests, or summary of inconsistency estimates from different parts of the treatment network. | **Supplementary 7;**  **6-7** |
| Risk of bias across studies | 22 | Present results of any assessment of risk of bias across studies for the evidence base being studied. | **Supplementary 13;**  **7** |
| Results of additional analyses | 23 | Give results of additional analyses, if done (e.g., sensitivity or subgroup analyses, meta-regression analyses*, alternative network geometries studied, alternative choice of prior distributions for Bayesian analyses,* and so forth). | **Supplementary 12;**  **7** |
|  |  |  |  |
| **DISCUSSION** |  |  |  |
| Summary of evidence | 24 | Summarize the main findings, including the strength of evidence for each main outcome; consider their relevance to key groups (e.g., healthcare providers, users, and policy-makers). | **7-8,10-12** |
| Limitations | 25 | Discuss limitations at study and outcome level (e.g., risk of bias), and at review level (e.g., incomplete retrieval of identified research, reporting bias). *Comment on the validity of the assumptions, such as transitivity and consistency. Comment on any concerns regarding network geometry (e.g., avoidance of certain comparisons).* | **12** |
| Conclusions | 26 | Provide a general interpretation of the results in the context of other evidence, and implications for future research. | **12-13** |
|  |  |  |  |
| **FUNDING** |  |  |  |
| Funding | 27 | Describe sources of funding for the systematic review and other support (e.g., supply of data); role of funders for the systematic review. This should also include information regarding whether funding has been received from manufacturers of treatments in the network and/or whether some of the authors are content experts with professional conflicts of interest that could affect use of treatments in the network. | **13** |

PICOS = population, intervention, comparators, outcomes, study design.

* Text in italics indicateS wording specific to reporting of network meta-analyses that has been added to guidance from the PRISMA statement.

† Authors may wish to plan for use of appendices to present all relevant information in full detail for items in this section.

**Supplementary 2. Search strategy**

Table S2. Search strategy used in each database

| Database | Search strategy |
| --- | --- |
| Web of Science | #1 TS=Chronic Fatigue Syndrome* or Fatigue Syndrome, Chronic or Chronic Fatigue Fibromyalgia Syndrom* or Chronic Fatigue Disorder* or Fatigue Disorder, Chronic or Systemic Exertion Intolerance Disease or Myalgic Encephalomyelitis or Postviral Fatigue Syndrom* or Fatigue Syndrome, Postviral or Syndrome, Postviral Fatigue  #2 TS= clinical trial OR randomized controlled trial OR controlled clinical trial  #3 TS=Medicine, Chinese Traditional or TCM or cupping therapy or cupping or Massaging or massage or tui na or naprapathy or Moxibustion or Acupuncture or Acupuncture Therapy or Dry Needling or Electroacupuncture or Acupuncture, Ear or needl* or auricular acupressure or ear pressure beans or auricular point sticking or scraping or ironing or plaster or acupoint application or Tai Ji or Tai chi or Qigong or Baduanjin or Taijiquan or chi kung or Wuqinxi or Liuzijue or Yijinjing or aromatherapy or Music Therapy or acupoint or acupoint injection or Embed* or meridian* or Complementary Therapy* or Alternative therapy  #1 and #2 and #3 |
| PubMed | #1 (((((((((((Chronic Fatigue Syndrome*[Title/Abstract]) OR (Fatigue Syndrome*, Chronic[Title/Abstract])) OR (Chronic Fatigue Fibromyalgia Syndrom*[Title/Abstract])) OR (Chronic Fatigue Disorde*[Title/Abstract])) OR (Fatigue Disorder, Chronic[Title/Abstract])) OR (Systemic Exertion Intolerance Disease[Title/Abstract])) OR (Myalgic Encephalomyelitis[Title/Abstract])) OR (Postviral Fatigue Syndrom*[Title/Abstract])) OR (Fatigue Syndrome, Postviral[Title/Abstract])) OR (Syndrome, Postviral Fatigue[Title/Abstract])) OR ("Fatigue Syndrome, Chronic"[Mesh])  #2((Medicine, Chinese Traditional[MeSH Terms] OR cupping therapy[Title/Abstract] OR cupping therapy[MeSH] OR cupping[Title/Abstract] OR cupping therapy[Title/Abstract] OR Massage[MeSH Terms] OR Massaging[Title/Abstract] OR massage[Title/Abstract] OR tui na[Title/Abstract] OR naprapathy[Title/Abstract]) OR (Moxibustion[MeSH Terms] OR Moxibustion[Title/Abstract] OR Acupuncture[MeSH Terms] OR Acupuncture Therapy[MeSH Terms] OR Dry Needling[MeSH Terms] OR Acupuncture[Title/Abstract] OR Electroacupuncture[Title/Abstract] OR Acupuncture, Ear[Title/Abstract] OR needle [Title/Abstract] OR Needling[Title/Abstract] OR auricular acupressure[Title/Abstract] OR ear pressure beans[Title/Abstract] OR auricular point sticking [Title/Abstract] OR "Acupuncture, Ear"[Mesh] OR scraping[Title/Abstract] OR ironing[Title/Abstract] OR plaster[Title/Abstract] OR acupoint application[Title/Abstract])) OR (Tai Ji[Mesh] OR "Qigong"[Mesh] OR Baduanjin[Title/Abstract] OR Taijiquan[Title/Abstract] OR Tai chi[Title/Abstract] OR Tai ji[Title/Abstract] OR Qigong[Title/Abstract] OR chi kung[Title/Abstract] OR Wuqinxi[Title/Abstract] OR Liuzijue [Title/Abstract] OR Yijinjing[Title/Abstract] OR Aromatherapy[Mesh] OR aromatherapy[Title/Abstract] OR Music Therapy[Mesh] OR Music Therapy[Title/Abstract] OR acupoint[Title/Abstract] OR acupoint injection[Title/Abstract] OR Embedding[Title/Abstract] OR meridian*[Title/Abstract] OR Complementary Therapy[Title/Abstract] OR alternative therapy[Title/Abstract]) OR "Complementary Therapies"[Mesh])  #1 AND #2 |
| Cochrane Library | #1 MeSH descriptor: [Fatigue Syndrome, Chronic] explode all trees  #2 (Chronic Fatigue Syndrome* or Fatigue Syndrome*, Chronic or Chronic Fatigue Fibromyalgia Syndrom* or Chronic Fatigue Disorder* or Fatigue Disorder, Chronic or Systemic Exertion Intolerance Disease or Myalgic Encephalomyelitis or Postviral Fatigue Syndrom* or Fatigue Syndrome, Postviral or Syndrome, Postviral Fatigue):ti,ab,kw  #3 #1 OR #2  #4 MeSH descriptor: [Medicine, Chinese Traditional] explode all trees  #5 MeSH descriptor: [Cupping Therapy] explode all trees  #6 MeSH descriptor: [Massage] explode all trees  #7 MeSH descriptor: [Moxibustion] explode all trees  #8 MeSH descriptor: [Acupuncture Therapy] explode all trees  #9 MeSH descriptor: [Acupuncture] explode all trees  #10 MeSH descriptor: [Dry Needling] explode all trees  #11 MeSH descriptor: [Acupuncture, Ear] explode all trees  #12 MeSH descriptor: [Tai Ji] explode all trees  #13 MeSH descriptor: [Qigong] explode all trees  #14 MeSH descriptor: [Music Therapy] explode all trees  #15 (Medicine, Chinese Traditional or TCM or cupping therapy or cupping or Massaging or massage or tui na or naprapathy or Moxibustion or Acupuncture or Acupuncture Therapy or Dry Needling or Electroacupuncture or Acupuncture, Ear or needl* or auricular acupressure or ear pressure beans or auricular point sticking or scraping or ironing or plaster or acupoint application or Tai Ji or Taichi or Qigong or Baduanjin or Taijiquan or chi kung or Wuqinxi or Liuzijue or Yijinjing or aromatherapy or Music Therapy or acupoint or acupoint injection or Embed* or meridian* or Complementary Therapy* or Alternative therapy):ti,ab,kw  #16 #4 or #5 or #6 or #7 or #8 or #9 or #10 or #11 or #12 or #13 or #14  #17 #15 or #16  #18 #3 and #17 |
| Scopus(506) | [TITLE-ABS-KEY ( "Chronic Fatigue Syndrome" OR "Fatigue Syndromes, Chronic" OR "Chronic Fatigue Fibromyalgia Syndrome" OR "Chronic Fatigue Disorder" OR "Fatigue Disorder, Chronic" OR "Systemic Exertion Intolerance Disease" OR "Myalgic Encephalomyelitis" OR "Postviral Fatigue Syndrome" OR "Fatigue Syndrome, Postviral" OR "Syndrome, Postviral Fatigue" ) ) AND [TITLE-ABS-KEY ( "Medicine, Chinese Traditional" OR "TCM" OR "cupping therapy" OR cupping OR Massaging OR massage OR "tui na" OR naprapathy OR Moxibustion OR Acupuncture OR "Acupuncture Therapy" OR "Dry Needling" OR Electroacupuncture OR "Acupuncture, Ear" OR needle OR "auricular acupressure" OR "ear pressure beans" OR "auricular point sticking" OR scraping OR ironing OR plaster OR "acupoint application" OR "Tai Ji" OR "Tai chi" OR Qigong OR Baduanjin OR Taijiquan OR "chi kung" OR Wuqinxi OR Liuzijue OR Yijinjing OR aromatherapy OR "Music Therapy" OR acupoint OR "acupoint injection" OR Embedding OR meridian OR "Complementary Therapy" OR "Alternative therapy" ) ) |
| CINHAL Complete via EBSCO | #1 (TI "Chronic Fatigue Syndrome*" OR AB "Chronic Fatigue Syndrome*" OR TI "Fatigue Syndrome, Chronic" OR AB "Fatigue Syndrome, Chronic" OR TI "Chronic Fatigue Fibromyalgia Syndrom*" OR AB "Chronic Fatigue Fibromyalgia Syndrom*" OR TI "Chronic Fatigue Disorde*" OR AB "Chronic Fatigue Disorde*" OR TI "Fatigue Disorder, Chronic" OR AB "Fatigue Disorder, Chronic" OR TI "Systemic Exertion Intolerance Disease" OR AB "Systemic Exertion Intolerance Disease" OR TI "Myalgic Encephalomyelitis" OR AB "Myalgic Encephalomyelitis" OR TI "Postviral Fatigue Syndrom*" OR AB "Postviral Fatigue Syndrom*" OR TI "Fatigue Syndrome, Postviral" OR AB "Fatigue Syndrome, Postviral" OR TI "Syndrome, Postviral Fatigue" OR AB "Syndrome, Postviral Fatigue") OR (MH "Fatigue Syndrome, Chronic")  #2 (MH "Chinese Medicine, Traditional+" OR TI "cupping therapy" OR AB "cupping therapy" OR MH "Cupping" OR TI "cupping" OR AB "cupping" OR MH "Massage+" OR TI "Massaging" OR AB "Massaging" OR TI "massage" OR AB "massage" OR TI "tui na" OR AB "tui na" OR TI "naprapathy" OR AB "naprapathy") OR (MH "Moxibustion" OR TI "Moxibustion" OR AB "Moxibustion" OR MH "Acupuncture+" OR MH "Acupuncture Therapy" OR MH "Dry Needling" OR TI "Acupuncture" OR AB "Acupuncture" OR TI "Electroacupuncture" OR AB "Electroacupuncture" OR TI "Acupuncture, Ear" OR AB "Acupuncture, Ear" OR TI "needle" OR AB "needle" OR TI "Needling" OR AB "Needling" OR TI "auricular acupressure" OR AB "auricular acupressure" OR TI "ear pressure beans" OR AB "ear pressure beans" OR TI "auricular point sticking" OR AB "auricular point sticking" OR MH "Ear, Acupuncture" OR TI "scraping" OR AB "scraping" OR TI "ironing" OR AB "ironing" OR TI "plaster" OR AB "plaster" OR TI "acupoint application" OR AB "acupoint application") OR (MH "Tai Ji" OR MH "Qi Gong" OR TI "Baduanjin" OR AB "Baduanjin" OR TI "Taijiquan" OR AB "Taijiquan" OR TI "Tai chi" OR AB "Tai chi" OR TI "Tai ji" OR AB "Tai ji" OR TI "Qigong" OR AB "Qigong" OR TI "chi kung" OR AB "chi kung" OR TI "Wuqinxi" OR AB "Wuqinxi" OR TI "Liuzijue" OR AB "Liuzijue" OR TI "Yijinjing" OR AB "Yijinjing" OR MH "Aromatherapy" OR TI "aromatherapy" OR AB "aromatherapy" OR MH "Music Therapy+" OR TI "Music Therapy" OR AB "Music Therapy" OR TI "acupoint" OR AB "acupoint" OR TI "acupoint injection" OR AB "acupoint injection" OR TI "Embedding" OR AB "Embedding" OR TI "meridian*" OR AB "meridian*" OR TI "Complementary Therapy" OR AB "Complementary Therapy" OR TI "alternative therapy" OR AB "alternative therapy") OR (MH "Complementary Therapies+")  #3 1 AND 2 |
| Embase(674) | #1 'chronic fatigue syndrome*':ab,ti OR 'fatigue syndrome*, chronic':ab,ti OR 'chronic fatigue fibromyalgia syndrom*':ab,ti OR 'chronic fatigue disorde*':ab,ti OR 'fatigue disorder, chronic':ab,ti OR 'systemic exertion intolerance disease':ab,ti OR 'myalgic encephalomyelitis':ab,ti OR 'postviral fatigue syndrom*':ab,ti OR 'fatigue syndrome, postviral':ab,ti OR 'syndrome, postviral fatigue':ab,ti  #2 'chronic fatigue syndrome'/exp OR 'myalgic encephalomyelitis'/exp OR 'postviral fatigue syndrome'/exp  #3 #1 OR #2  #4 'traditional chinese medicine'/exp OR 'acupuncture'/exp OR 'electroacupuncture'/exp OR 'moxibustion'/exp OR 'cupping therapy'/exp OR 'massage'/exp OR 'qigong'/exp OR 'tai chi'/exp OR 'aromatherapy'/exp OR 'music therapy'/exp OR 'complementary therapy'/exp  #5 cupping:ti,ab,kw OR 'cupping therapy':ti,ab,kw OR massage:ti,ab,kw OR massaging:ti,ab,kw OR 'tui na':ti,ab,kw OR naprapathy:ti,ab,kw  #6 moxibustion:ti,ab,kw OR acupuncture:ti,ab,kw OR electroacupuncture:ti,ab,kw OR 'dry needling':ti,ab,kw OR 'ear acupuncture':ti,ab,kw OR 'auricular acupressure':ti,ab,kw OR 'ear pressure beans':ti,ab,kw OR 'auricular point sticking':ti,ab,kw OR scraping:ti,ab,kw OR ironing:ti,ab,kw OR plaster:ti,ab,kw OR 'acupoint application':ti,ab,kw  #7 'tai chi':ti,ab,kw OR 'tai ji':ti,ab,kw OR taijiquan:ti,ab,kw OR qigong:ti,ab,kw OR 'chi kung':ti,ab,kw OR baduanjin:ti,ab,kw OR wuqinxi:ti,ab,kw OR aromatherapy:ti,ab,kw OR 'music therapy':ti,ab,kw OR acupoint:ti,ab,kw OR 'acupoint injection':ti,ab,kw OR embedding:ti,ab,kw OR meridian*:ti,ab,kw OR 'complementary therapy':ti,ab,kw OR 'alternative therapy':ti,ab,kw OR 'complementary therapies liuzijue':ti,ab,kw OR yijinjing:ti,ab,kw  #8 #4 OR #5 OR #6 OR #7  #9 #3 AND #8 |
| China National Knowledge Infrastructure Database | SU%=慢性疲劳综合征 + 慢性疲劳综合症 + 肌痛性脑脊髓炎 + 慢性疲劳纤维肌痛综合征 + 慢性疲劳和免疫功能障碍综合征 AND SU%=针刺 + 针 + 穴位埋线 + 艾灸 + 灸 + 耳穴 + 耳穴压豆 + 芳香疗法 + 芳香 + 音乐疗法 + 音乐 + 五音疗法 + 刮痧 + 推拿 + 按摩 + 穴位 + 穴 + 敷 + 外敷 + 熏 + 浴 + 熨 + 罐 + 拔罐 + 走罐 + 火罐 + 气功 + 太极拳 + 五禽戏 + 八段锦 + 六字诀 + 易筋经 AND SU%=随机对照试验 + 临床观察 + 临床试验 + 临床研究 + 疗效 + 观察 + 随机对照 NOT SU%=大鼠 + 小鼠 |
| Wanfang | 题名或关键词:(慢性疲劳综合征 OR 慢性疲劳综合症 OR 肌痛性脑脊髓炎 OR 慢性疲劳纤维肌痛综合征 OR 慢性疲劳和免疫功能障碍综合征) and 题名或关键词:(针刺 OR 针 OR 穴位埋线 OR 艾灸 OR 灸 OR 耳穴 OR 耳穴压豆 OR 芳香疗法 OR 芳香 OR 音乐疗法 OR 音乐 OR 五音疗法 OR 刮痧 OR 推拿 OR 按摩 OR 穴位 OR 穴 OR 敷 OR 外敷 OR 熏 OR 浴 OR 熨 OR 罐 OR 拔罐 OR 走罐 OR 火罐 OR 气功 OR 太极拳 OR 五禽戏 OR 八段锦 OR 六字诀 OR 易筋经) and (随机对照试验 OR 临床观察 OR 临床试验 OR 临床研究 OR 疗效 OR 观察) not (大鼠 OR 小鼠 OR 系统评价 OR 研究进展 OR Meta分析) |
| Sinomed | #1 "针刺疗法"[不加权:扩展]) OR "针灸疗法"[不加权:扩展]) OR "耳穴疗法"[不加权:扩展])) OR "芳香疗法"[不加权:扩展]) OR "音乐疗法"[不加权:扩展]) OR "刮痧疗法"[不加权:扩展]) OR "推拿疗法"[不加权:扩展]) OR "药膳疗法"[不加权:扩展]) OR "穴位贴敷疗法"[不加权:扩展] OR "穴位埋线疗法"[不加权:扩展] OR "水针疗法"[不加权:扩展]) OR "熏洗疗法"[不加权:扩展]) OR "药熨疗法"[不加权:扩展]  #2 "针刺"[常用字段:智能] OR "针"[常用字段:智能] OR "穴位埋线"[常用字段:智能] OR "艾灸"[常用字段:智能] OR "灸"[常用字段:智能] OR "耳穴"[常用字段:智能] OR "耳穴压豆"[常用字段:智能] OR "芳香疗法"[常用字段:智能] OR "芳香"[常用字段:智能] OR "音乐疗法"[常用字段:智能] OR "音乐"[常用字段:智能] OR "五音疗法"[常用字段:智能] OR "刮痧"[常用字段:智能] OR "推拿"[常用字段:智能] OR "按摩"[常用字段:智能] OR "药膳"[常用字段:智能] OR "穴位"[常用字段:智能] OR "穴"[常用字段:智能] OR "敷"[常用字段:智能] OR "外敷"[常用字段:智能] OR "熏"[常用字段:智能] OR " "[常用字段:智能] OR "熨"[常用字段:智能]  #3 "疲劳综合征, 慢性"[不加权:扩展]  #4 ("慢性疲劳综合征"[常用字段:智能] OR "慢性疲劳综合症"[常用字段:智能] OR "肌痛性脑脊髓炎"[常用字段:智能] OR "慢性疲劳纤维肌痛综合征"[常用字段:智能] OR "慢性疲劳和免疫功能障碍综合征"[常用字段:智能])  #5 #1 OR #2  #6 #3 OR #4  #7 #5 AND #6 |
| VIP Database | (M=(慢性疲劳综合征 OR 慢性疲劳综合症 OR 肌痛性脑脊髓炎 OR 慢性疲劳纤维肌痛综合征 OR 慢性疲劳和免疫功能障碍综合征) AND M=(针刺 OR 针 OR 穴位埋线 OR 艾灸 OR 灸 OR 耳穴 OR 耳穴压豆 OR 芳香疗法 OR 芳香 OR 音乐疗法 OR 音乐 OR 五音疗法 OR 刮痧 OR 推拿 OR 按摩 OR 穴位 OR 穴 OR 敷 OR 外敷 OR 熏 OR 浴 OR 熨 OR 罐 OR 拔罐 OR 走罐 OR 火罐 OR 气功 OR 太极拳 OR 五禽戏 OR 八段锦 OR 六字诀 OR 易筋经) AND U=(随机对照试验 OR 临床观察 OR 临床试验 OR 临床研究 OR 疗效 OR 观察) |

**Supplementary 3. Additional details on Methods**

Table S3. Definitions of Each Intervention and Control

| Nodes | Definition of Intervention |
| --- | --- |
| Moxibustion | Moxibustion is an external herbal treatment used in traditional Chinese medicine. It works by burning mugwort fluff made from dried mugwort leaves (also known as Chinese herbal mugwort) to produce a thermal effect on specific acupoints, thereby providing therapeutic benefits purposes^26^. |
| Acupuncture | Acupuncture is based on Traditional Chinese Medicine meridian theory. It involves inserting fine metal needles into acupoints and stimulating them through electrical, manual, or other methods stimulation^27^. Studies that did not use needles or involved nonmanual needle stimuli, including electroacupuncture, were excluded. |
| Massage | Massage is a therapeutic technique that emphasizes stimulating specific body areas to regulate related or overall physiological functions, thereby achieving therapeutic benefits goals^28^. |
| Qigong | Qigong, guided by fundamental theories of traditional Chinese medicine, achieves harmony and unity of the body, breath, and spirit through regulating physical movements, breathing, and mental focus, thereby strengthening the body, preventing illness, and treating disease^29^. |
| Cupping | Cupping therapy usually uses glass, bamboo, or silicone cups to create suction on the skin. The cup is placed on the skin, and the air inside is heated and then cooled, forming a vacuum. This suction pulls the skin upward into the cup, boosting blood flow in the area^30^. |
| Acupoint application | Acupoint plaster therapy is a non-invasive treatment based on Traditional Chinese Medicine meridian theory. It uses transdermal drug absorption principles on the body's surface to provide therapy effects^31^. |
| Scraping | Scraping therapy is an external TCM treatment. By scraping the skin, stagnant qi is moved from the interior to the exterior, rapidly promoting the circulation of qi and blood throughout the body to achieve a therapeutic effect^32^. |
| Inert Treatment | Sham therapy^33^,This article primarily discusses sham acupuncture, which is often used as a control. However, it must be recognized that it may not be entirely ineffective; therefore, sham acupuncture is considered an independent node. |
| Conventional care | Include but not restrict to Usual care, No treatment/waiting list, Non-specific symptomatic drugs (as used in CFS), General advice (including education, reassurance, exercise, and bed rest)^33^. |

**Supplementary 4. Risk of bias assessment for the included studies**

Table S4. Risk of bias assessment for the included studies

| Study | Randomization  process | Deviations  from  intended  interventions | Missing  outcome  data | Measurement  of the outcome | Selection of  the reported  result | Overall quality |
| --- | --- | --- | --- | --- | --- | --- |
| Peng ZC,2024 | Low | High | Low | Some concerns | Low | High |
| Lin YF,2021 | Low | Some concerns | Low | Low | Low | Some concerns |
| Li ZJ,2024 | Some concerns | Some concerns | Some concerns | Low | Low | Some concerns |
| Wang HN,2016 | Some concerns | High | Low | Low | Low | High |
| Wang JJ,2009 | Low | Some concerns | Low | Low | Low | Some concerns |
| Liang H,2017 | Some concerns | Some concerns | Low | Low | Low | Some concerns |
| Chen XH,2010 | Low | Some concerns | Low | Some concerns | Low | Some concerns |
| Ma J,2022 | Some concerns | High | Low | Some concerns | Some concerns | High |
| J.S.Chan,2013 | Some concerns | Low | Low | Low | Low | Some concerns |
| Yu JY,2019 | Some concerns | High | Low | Some concerns | Low | High |
| J.S.CHAN,2014 | Some concerns | Low | Low | Low | Low | Some concerns |
| Li HN,2017 | Some concerns | Some concerns | Low | Low | Low | Some concerns |
| Rainbow T H,2012 | Some concerns | Low | Low | High | Low | High |
| Ma S,2022 | Some concerns | Some concerns | High | High | Some concerns | High |
| Gong Y,2021 | Some concerns | High | Low | High | Low | High |
| Shang K,2019 | High | High | Low | Low | Low | High |
| Ma J,2018 | Some concerns | High | Low | High | Low | High |
| Hu Q,2016 | Some concerns | Some concerns | Low | High | Low | High |
| Tian L,2015 | Some concerns | High | Low | Low | Low | High |
| Cheng XH,2014 | Some concerns | High | Low | High | Low | High |
| Zhang W, 2010 | Some concerns | Some concerns | Low | High | Low | High |
| Liu J,2022 | High | High | Low | Some concerns | Some concerns | High |
| Liang WL, 2018 | Some concerns | High | Low | High | Low | High |
| Siu-Man Ng,2013 | Some concerns | Low | Some concerns | Low | Low | Some concerns |
| Chen XH,2011 | Some concerns | High | Low | Some concerns | Low | High |
| Xu YX,2019 | Some concerns | Some concerns | Low | Low | Low | Some concerns |
| Xu YX,2018 | Some concerns | Some concerns | Low | Some concerns | Low | Some concerns |
| Dou FH,2023 | High | High | Low | High | Some concerns | High |
| Dogukan kurc,2025 | High | Some concerns | Some concerns | High | Low | High |

Figure S3. Risk of Bias Summary for All Included Studies
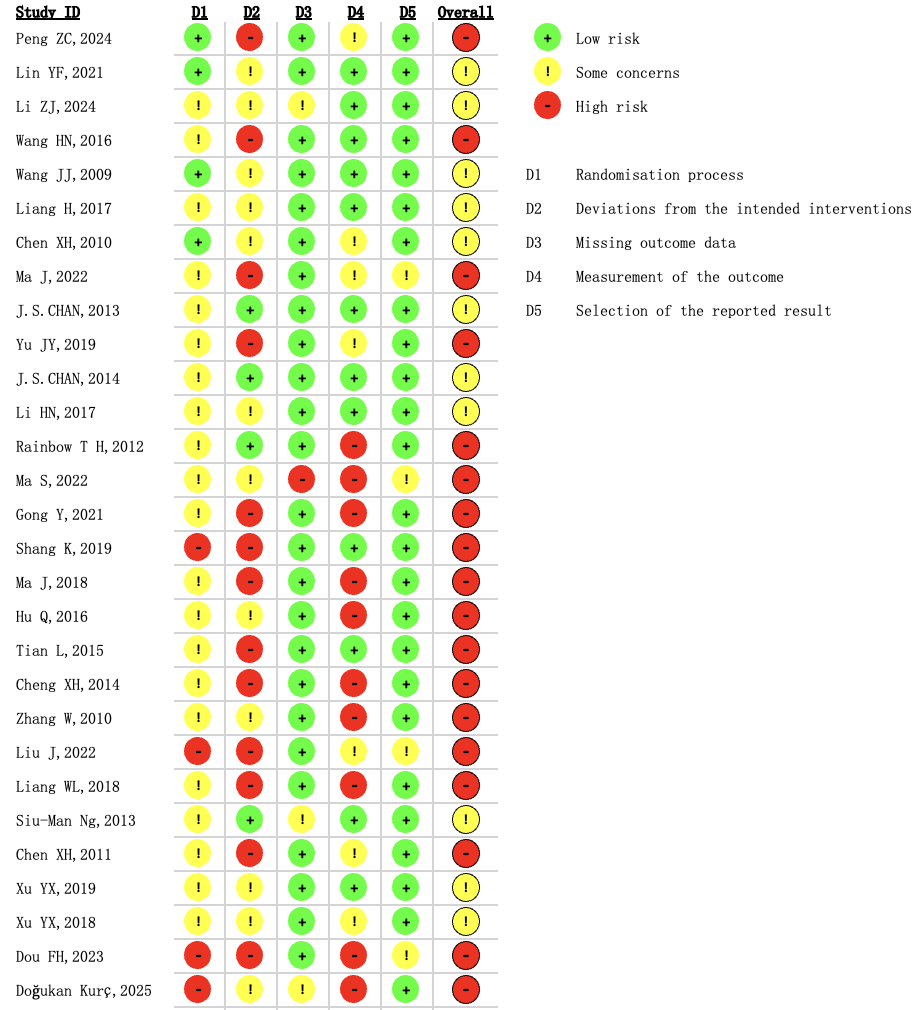


**Supplementary 5. Pairwise meta-analyses**

5.1. Pairwise meta-analyses: Overall Fatigue

Table S5.1. Pairwise meta-analyses for Overall Fatigue

| Comparison | Number of studies | Number of patients | SMD (95% CI) | P-value for the effect estimates | I^2^ (%) |
| --- | --- | --- | --- | --- | --- |
| Acupuncture vs Conventional care | 2 | 150 | **-1.01 [-1.36, -0.67]** | **0.00** | 0.0 |
| Moxibustion vs Conventional care | 3 | 237 | **-2.02 [-2.91, -1.12]** | **0.00** | 86.9 |
| Massage vs Conventional care | 5 | 359 | **-1.02 [-1.42, -0.63]** | **0.00** | 67.0 |
| Scraping vs Conventional care | 2 | 120 | **-0.81 [-1.19, -0.44]** | **0.00** | 0.0 |
| Qigong vs Conventional care | 4 | 351 | **-0.85 [-1.16, -0.55]** | 0.00 | 45.8 |
| Cupping vs Conventional care | 1 | 60 | **-0.91 [-1.44, -0.38]** | **0.00** | NA |
| Acupuncture vs Inert treatment | 2 | 109 | -0.68 [-1.45, 0.09] | 0.09 | 73.3 |
| Moxibustion vs Acupuncture | 3 | 208 | **-0.91 [-1.20, -0.63]** | **0.00** | 33.7 |
| Massage vs Acupuncture | 2 | 137 | **-0.58 [-0.93, -0.24]** | **0.00** | 62.7 |
| Acupoint application vs Acupuncture | 1 | 60 | -0.39**[**-0.91, 0.12**]** | 0.13 | NA |

5.2.Pairwise meta-analyses: Physical Fatigue

Table S5.2 Pairwise meta-analyses for Physical Fatigue

| Comparison | Number of studies | Number of patients | SMD (95% CI) | P-value for the effect estimates | I^2^ (%) |
| --- | --- | --- | --- | --- | --- |
| Acupuncture vs Conventional care | 3 | 223 | **-1.65 [-1.96, -1.35]** | **0.00** | 0.0 |
| Massage vs Conventional care | 2 | 134 | **-2.24 [-2.67, -1.80]** | **0.00** | 0.0 |
| Qigong vs Conventional care | 3 | 351 | **-1.07 [-1.41, -0.72]** | **0.00** | 54.4 |
| Cupping vs Conventional care | 1 | 60 | **-0.88 [-1.41, -0.35]** | **0.00** | NA |
| Acupuncture vs Inert treatment | 2 | 163 | **-0.37 [-0.68, -0.06]** | **0.02** | 0 |
| Moxibustion vs Acupuncture | 1 | 60 | -0.50[-1.02, 0.01] | 0.06 | NA |
| Acupoint application vs Acupuncture | 1 | 60 | -0.24 [-0.75, 0.27] | 0.35 | NA |

5.3. Pairwise meta-analyses: Mental Fatigue

Table S5.3 Pairwise meta-analyses for Mental Fatigue

| Comparison | Number of studies | Number of patients | SMD (95% CI) | P-value for the effect estimates | I^2^ (%) |
| --- | --- | --- | --- | --- | --- |
| Acupuncture vs Conventional care | 3 | 223 | **-1.24 [-2.43, -0.05]** | **0.04** | 93.7 |
| Massage vs Conventional care | 2 | 134 | **-2.09 [-3.60, -0.58]** | **0.01** | 91.7 |
| Qigong vs Conventional care | 3 | 351 | **-0.54 [-0.77, -0.30]** | **0.00** | 14.2 |
| Cupping vs Conventional care | 1 | 60 | -0.35 [-0.86, 0.16] | 0.17 | NA |
| Acupuncture vs Inert treatment | 2 | 163 | -0.53 [-0.84, -0.21] | **0.00** | 0 |
| Moxibustion vs Acupuncture | 1 | 60 | -0.53[-1.05,-0.02] | **0.04** | NA |
| Acupoint application vs Acupuncture | 1 | 60 | -0.07 [-0.57, 0.44] | 0.80 | NA |

5.4. Pairwise meta-analyses: Sleep Quality

Table S5.4 Pairwise meta-analyses for Sleep Quality

| Comparison | Number of studies | Number of patients | SMD [95% CI] | P-value for the effect estimates | I^2^ (%) |
| --- | --- | --- | --- | --- | --- |
| Massage vs Conventional care | 2 | 139 | -4.46 [-5.40,-3.52] | **0.00** | 0.0 |
| Cupping vs Conventional care | 1 | 60 | -4.60 [-5.92, -3.28] | **0.00** | NA |
| Qigong vs Conventional care | 1 | 150 | -1.10 [-2.27, 0.07] | 0.07 | NA |
| Scraping vs Conventional care | 1 | 60 | -1.47 [-2.69, -0.25] | **0.02** | NA |
| Moxibustion vs Conventional care | 3 | 210 | -2.92 [-4.39, -1.45] | **0.00** | 82.3 |
| Acupuncture vs Conventional care | 1 | 60 | -4.60 [-5.85, -3.35] | **0.00** | NA |

5.5. Pairwise meta-analyses: Anxiety

Table S5.5 Pairwise meta-analyses for Anxiety

| Comparison | Number of studies | Number of patients | SMD (95% CI) | P-value for the effect estimates | I^2^ (%) |
| --- | --- | --- | --- | --- | --- |
| Acupuncture vs Conventional care | 2 | 150 | -2.08 [-6.04, 1.87] | 0.30 | 98.4 |
| Qigong vs Conventional care | 2 | 287 | -0.28 [-0.56, 0.01] | 0.06 | 32.7 |
| Moxibustion vs Acupuncture | 2 | 137 | **-1.33 [-2.51, -0.16]** | **0.03** | 89.2 |

5.6. Pairwise meta-analyses: Depression

Table S5.6 Pairwise meta-analyses for Depression

| Comparison | Number of studies | Number of patients | SMD (95% CI) | P-value for the effect estimates | I^2^ (%) |
| --- | --- | --- | --- | --- | --- |
| Moxibustion vs Conventional care | 1 | 108 | **-0.73 [-1.12, -0.34]** | **0.00** | NA |
| Qigong vs Conventional care | 2 | 287 | **-0.57 [-0.81, -0.34]** | **0.00** | 0.0 |
| Acupuncture vs Conventional care | 1 | 60 | **-1.21 [-1.77, -0.66]** | **0.00** | NA |
| Massage vs Conventional care | 1 | 39 | **-0.78 [-1.43, -0.12]** | **0.02** | NA |
| Moxibustion vs Acupuncture | 2 | 137 | **-0.53 [-0.87, -0.18]** | **0.00** | 29.0 |

Abbreviations: NA: Not Available.

**Supplementary 6. Assessment of transitivity**

Table S6 Study and Patient characteristics

| Study | Year | Diagnostic criteria | Mean Illness duration(month) | Baseline Fatigue severity（%） | Female(%) | Age(Year) | Intervention/Control | Sample Size | Lenth of intervention (Week) | Frequency | Outcome instrument |
| --- | --- | --- | --- | --- | --- | --- | --- | --- | --- | --- | --- |
| Peng ZC | 2024 | 1994CDC | NA | 66.90 | 43.8 | 38.8±16.5 | Massage | 48 | 4week | 12 | FAI |
|  |  |  | NA | 62.68 | 46.8 | 40.0±18.3 | Conventional care | 47 | 4week | 84 |  |
| Lin YF | 2021 | 1994CDC | 17±7 | 72.93 | 78.6 | 34±9 | Moxibustion | 28 | 4week | 12 | FS-14 |
|  |  |  | 17±7 | 71.21 | 82.6 | 35±9 | Conventional care | 29 | NA | NA |  |
| Li ZJ | 2024 | 1994CDC | 24.1±20.7 | 71.64 | 58.06 | 31±8 | Massage | 31 | 4week | 12 | FS-14, PSQI |
|  |  |  | 33.4±32.8 | 69.00 | 68.57 | 32±9 | Conventional care | 35 | NA | NA |  |
| Wang HN | 2016 | 1994CDC | 38.47± 31.59 | 71.79 | 48.7 | 36.49 ± 8.88 | Massage | 39 | NA | 10 | FSS, PSQI |
|  |  |  | 37.40± 28.30 | 71.25 | 50 | 37.32 ± 9.30 | Conventional care | 40 | NA | 10 |  |
| Wang JJ | 2009 | 1994CDC | NA | 70.71 | 59.4 | 35.8±10.7 | Acupuncture | 32 | 4week | 12 | FS-14 |
|  |  |  | NA | 68.57 | 59.4 | 38.8±8.8 | Inert treatment | 32 | 4week | 12 |  |
| Liang H | 2017 | 1994CDC | 3.81±1.23 | 61.21 | 63.33 | 41.16±8.28 | Acupuncture | 30 | 4week | 20 | FS-14, PSQI |
|  |  |  | 3.77±1.19 | 61.57 | 66.67 | 41.53±8.64 | Conventional care | 30 | 4week | 12 |  |
| Chen XH | 2010 | 1994CDC | 2.06±0.98 | 66.93 | 57.78 | 38.3±9.9 | Acupuncture | 45 | 2week | 14 | FS-14 |
|  |  |  | 3.17±1.23 | 64.86 | 53.3 | 40.6±9.5 | Conventional care | 45 | 2week | 14 |  |
| Ma J | 2022 | 1994CDC | 1.62±0.36 | 72.50 | 31.48 | 39.48±8.35 | Moxibustion | 54 | 4week | 12 | FS-14, SDS |
|  |  |  | 1.66±0.34 | 72.93 | 35.19 | 39.52±8.62 | Conventional care | 54 | 4week | 12 |  |
| J.S.Chan | 2013 | 1994CDC | 6.4±1.4 | 70.89 | 72.22 | 42.4±6.7 | Qigong | 72 | 17week | 60 | ChCFS,  HADS |
|  |  |  | 6.4±1.4 | 71.07 | 81.53 | 42.5±6.4 | Conventional care | 65 | NA | NA |  |
| Yu JY | 2019 | 1994CDC | 38.9±14.1 | 65.93 | 46.67 | 45.2±11.6 | Cupping | 30 | 4week | 14 | FS-14, PSQI |
|  |  |  | 35.1±16.0 | 70.00 | 43.33 | 43.5±12.7 | Conventional care | 30 | 4week | 28 |  |
| J.S.Chan | 2014 | 1994CDC | NA | 66.79 | 61.3 | 39.1±7.8 | Qigong | 75 | 9week | 16 | ChCFS, PSQI, HADS |
|  |  |  | NA | 65.00 | 82.7 | 38.9±8.1 | Conventional care | 75 | NA | NA |  |
| Rainbow T H Ho | 2012 | 1994CDC | NA | 71.25 | 75.8 | 42.1±7.3 | Qigong | 33 | 17week | 60 | ChCFS |
|  |  |  | NA | 70.89 | 83.6 | 42.5±5.5 | Conventional care | 31 | NA | NA |  |
| Li HN | 2017 | 1994CDC | 10.4±5.0 | 63.57 | 43.59 | 41.8±7.1 | Massage | 39 | 4week | 20 | FS-14, HAMD, SAS |
|  |  |  | 10.6±5.4 | 66.43 | 36.84 | 42.63±6.2 | Acupuncture | 38 | 4week | 20 |  |
| Ma S | 2022 | 1994CDC | NA | 69.29 | 53.3 | 37.23 ± 7.80 | Acupuncture | 30 | 4week | 24 | FS-14, SDS,  SAS |
|  |  |  | NA | 85.00 | 50 | 37.70 ± 8.36 | Conventional care | 30 | 4week | 24 |  |
| Gong Y | 2021 | 1994CDC | 27.25±5.62 | 61.21 | 63.3 | 42.85 ± 8.65 | Scraping | 30 | 4week | 5 | FS-14、PSQI |
|  |  |  | 26.84±6.05 | 61.50 | 60 | 41.64 ± 7.88 | Conventional care | 30 | 4week | 30 |  |
| Shang K | 2019 | 1994CDC | 2.4±1.4 | 76.98 | 51.4 | 31±9.4 | Massage | 35 | 3week | 21 | FAI |
|  |  |  | 2.6±1.3 | 75.60 | 54.3 | 30±8.8 | Conventional care | 35 | 4week | 30 |  |
| Ma J | 2018 | 1994CDC | NA | 80.19 | 57.9 | 43±6 | Moxibustion | 38 | 6week | 40 | FAI |
|  |  |  | NA | 81.34 | 47.4 | 43±7 | Acupuncture | 38 | 6week | 40 |  |
| Hu Q | 2016 | 1994CDC | NA | 52.14 | 43.3 | 35.14±3.51 | Massage | 30 | 6week | 20 | FS-14,  DSI,  SAS |
|  |  |  | NA | 54.29 | 50 | 36.14±4.23 | Acupuncture | 30 | 6week | 20 |  |
| Tian L | 2015 | 1994CDC | NA | 79.96 | 33.3 | 42±9 | Moxibustion | 36 | 4week | 30 | FAI |
|  |  |  | NA | 83.17 | 55.5 | 42±10 | Acupuncture | 36 | 4week | 30 |  |
| Cheng XH | 2014 | 1994CDC | 15. 67± 6. 34 | 61.64 | 53.3 | 31.33±5.94 | Acupoint application | 30 | 6week | 20 | FS-14 |
|  |  |  | 13. 30± 5. 81 | 54.29 | 56.7 | 31.30±5.68 | Acupuncture | 30 | 6week | 20 |  |
| Zhang W | 2010 | 1994CDC | ８.42±１.47 | 61.04 | 45.5 | 48.0±13.5 | Acupuncture | 22 | 4week | 20 | ChCFS |
|  |  |  | ８.86±１.47 | 65.98 | 47.8 | 43.2±12.4 | Inert treatment | 23 | 4week | 20 |  |
| Liu J | 2022 | 1994CDC | NA | 63.86 | 60 | 45.36±2.13 | Moxibustion | 30 | 12week | 48 | FS-14 |
|  |  |  | NA | 63.79 | 60.33 | 40.20±12.67 | Acupuncture | 30 | 12week | 48 |  |
| Liang WL | 2018 | 1994CDC | 9.21±1.61 | 67.07 | 60 | 40±9 | Moxibustion | 35 | 4week | 24 | FS-14 |
|  |  |  | 9.37±2.30 | 68.64 | 65.7 | 41±11 | Conventional care | 35 | 4week | 84 |  |
| Siu-Man Ng | 2013 | 1994CDC | NA | NA | 72 | 39.8±6.6 | Acupuncture | 50 | 4week | 8 | ChCFS |
|  |  |  | NA | NA | 65.3 | 42.0±6.5 | Inert treatment | 49 | 4week | 8 |  |
| Chen XH | 2011 | 1994CDC | 2.06±0.98 | NA | 57.8 | 38±10 | Acupuncture | 45 | 2week | 14 | SAS |
|  |  |  | 3.17±1.23 | NA | 53.3 | 41±10 | Conventional care | 45 | 2week | 14 |  |
| Xu YX | 2019 | 1994CDC | 11.7±4.2 | NA | 73.53 | 39±9 | Acupuncture | 34 | 4week | 20 | FS-14, PSQI |
|  |  |  | 10.9±4.5 | NA | 67.65 | 37±9 | Conventional care | 34 | 4week | 20 |  |
| Xu YX | 2018 | 1994CDC | NA | NA | 67.6 | NA | Massage | 37 | 4week | 12 | FS-14, PSQI |
|  |  |  | NA | NA | 72.2 | NA | Conventional care | 36 | 4week | 12 |  |
| Dou FH | 2023 | 1994CDC | 6.27±0.84 | NA | 60 | 47.46±1.93 | Moxibustion | 40 | 4week | 20 | PSQI |
|  |  |  | 6.33±0.89 | NA | 57.5 | 47.63±1.99 | Conventional care | 40 | 4week | 28 |  |
| Doğukan Kurç | 2025 | 1994CDC | NA | 48.14 | 70.6 | 26.64 ± 1.29 | Massage | 17 | 4week | 10 | FSS,BDI |
|  |  |  | NA | 62.29 | 72.7 | 29.54 ± 1.27 | Conventional care | 22 | NA | NA |  |

**Supplementary 7.** **Assessment of inconsistency**

7.1. Estimated global inconsistency in networks

Table S7.1. Estimated global inconsistency in networks

| **Primary outcomes** | Chi square | Prob > chi2 |
| --- | --- | --- |
| Overall Fatigue | chi2(2) = 2.08 | Prob > chi2 = 0.3529 |
| **Secondary Outcomes** | Chi square | Prob > chi2 |
| Physical Fatigue | chi2(1) = 0.79 | Prob > chi2 = 0.3728 |
| Mental Fatigue | chi2(1) = 1.97 | Prob > chi2 = 0.16 |
| Sleep Quality | chi2(1) = 2.72 | Prob > chi2 = 0.0989 |
| Depression | chi2(1) = 3.60 | Prob > chi2 = 0.0577 |
| Anxiety | chi2(1) = 0.05 | Prob > chi2 =0.8176 |

7.2 Estimated local inconsistency in networks

Figure S7.2a. Local inconsistency for Overall Fatigue


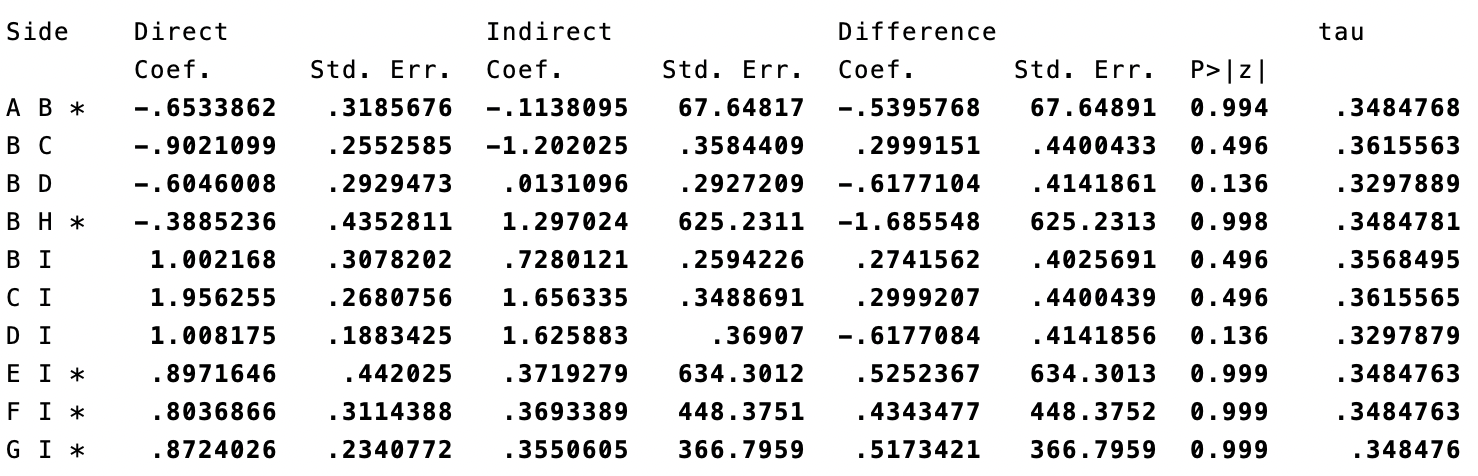


Abbreviations: A: Inert treatment, B: Acupuncture, C: Moxibustion, D: Massage, E: Cupping, F: Scraping, G: Qigong, H: Acupoint application, I: Conventional care.

Footnote: *All the evidence about these contrasts comes from the trials which directly compare them.

Figure S7.2b. Local inconsistency for Physical Fatigue


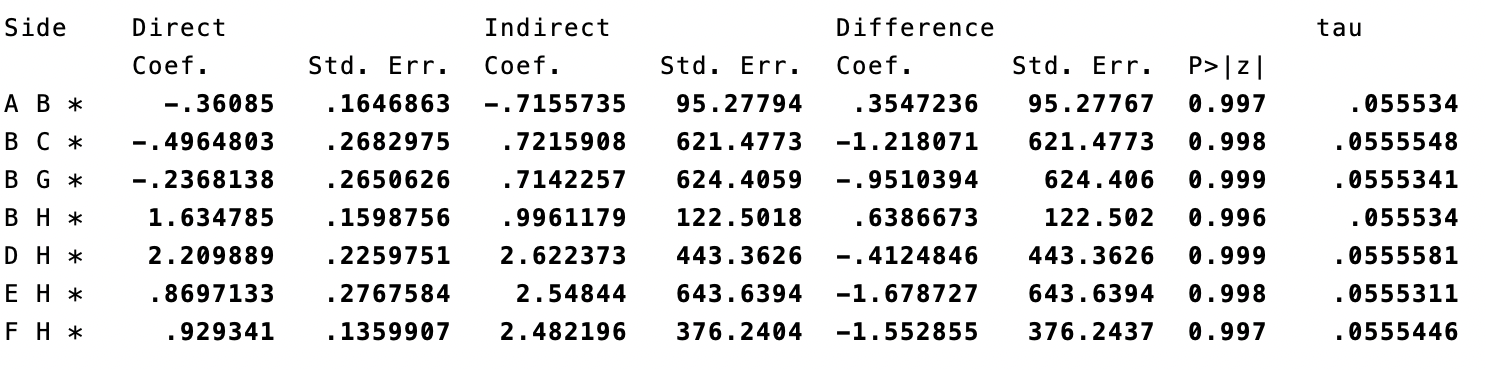


Abbreviations: A: Inert treatment, B: Acupuncture, C: Moxibustion, D: Massage, E: Cupping, F: Qigong, G: Acupoint application, H: Conventional care .

Footnote: *All the evidence about these contrasts comes from the trials which directly compare them.

Figure S7.2c. Local inconsistency for Mental Fatigue


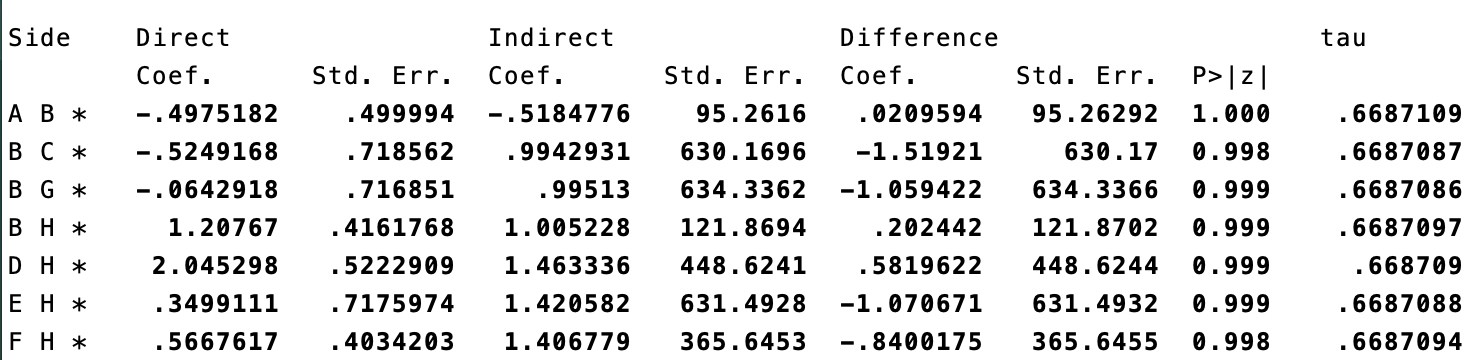


Abbreviations: A: Inert treatment, B: Acupuncture, C: Moxibustion, D: Massage, E: Cupping, F: Qigong, G: Acupoint application, H: Conventional care .

Footnote: *All the evidence about these contrasts comes from the trials which directly compare them.

Figure S7.2d. Local inconsistency for Sleep quality


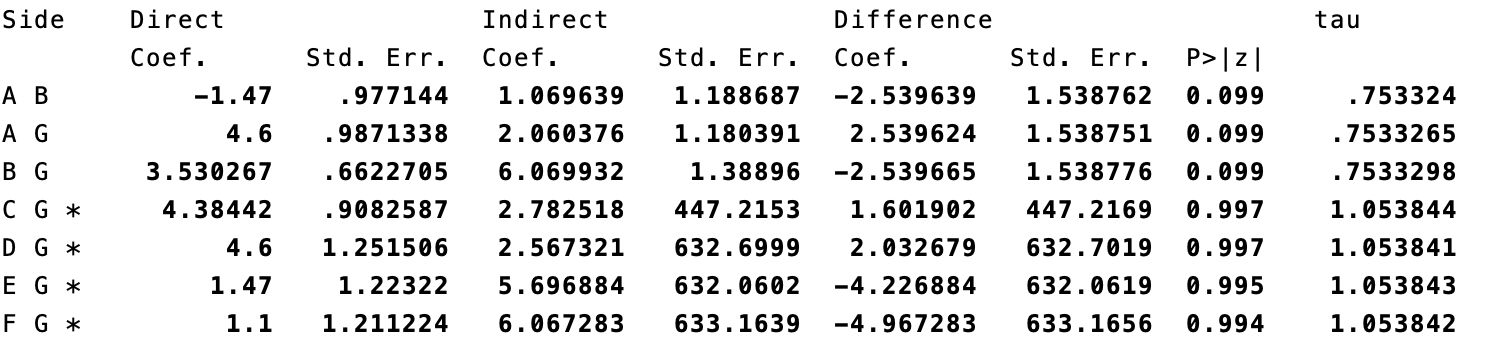


Abbreviations: A: Acupuncture, B: Moxibustion, C: Massage, D: Cupping, E:Scraping, F: Qigong, G: Conventional care.

Footnote: *All the evidence about these contrasts comes from the trials which directly compare them.

Figure S7.2e. Local inconsistency for Anxiety


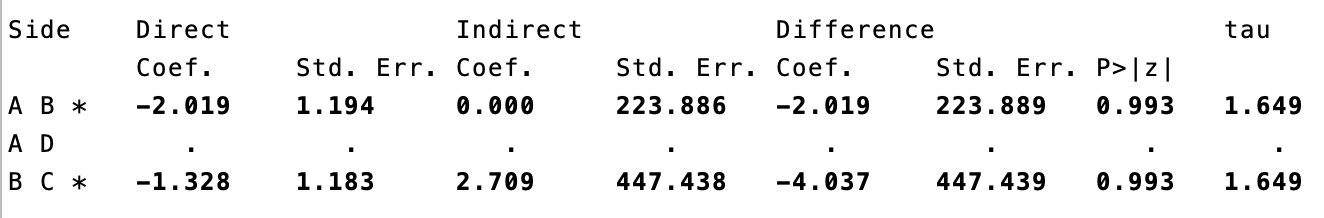


Abbreviations: A: Acupuncture, B: Massage, C: , D: Qigong, E: Conventional care.

Footnote: *All the evidence about these contrasts comes from the trials which directly compare them.

Figure S7.2f. Local inconsistency for Depression


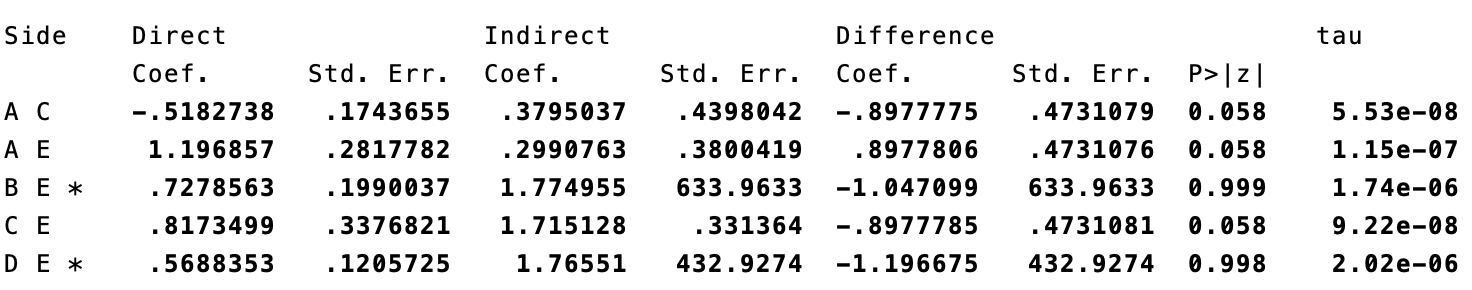


Abbreviations: A: Acupuncture, B: Moxibustion, C: Massage, D: Qigong, E: Conventional care.

Footnote: *All the evidence about these contrasts comes from the trials which directly compare them.

**Supplementary 8. Comparison-adjusted funnel plots**

Because sleep quality, depression, and anxiety were reported by less than 10 articles, it was not feasible to assess small study effects. There was no small-study effect based on the comparison adjusted funnel plots and the Egger test results for other outcomes.

**Figure S8 The funnel plot for Fatigue**

**
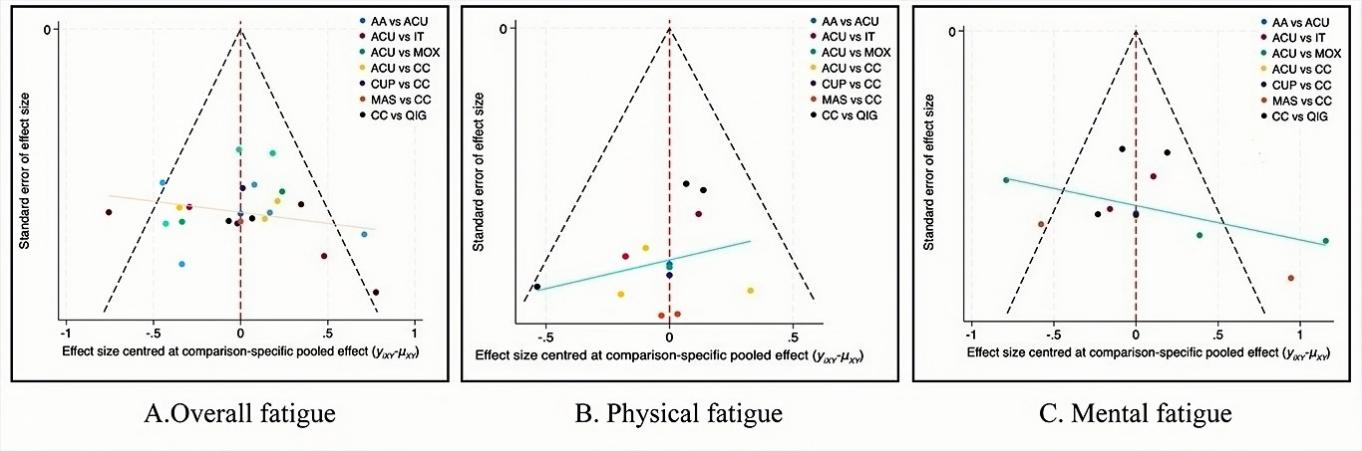
**

Figure S7.A The funnel plot for Overall Fatigue; Egger test: P = 0.06

Figure S7.B The funnel plot for Physical Fatigue; Egger test: P= 0.21

Figure S7.C The funnel plot for Mental Fatigue; Egger test: P= 0.22

Abbreviations: IT: Inert treatment, ACU: Acupuncture, MOX: Moxibustion, MAS: Massage, CUP: Cupping, QIG: Qigong, AA: Acupoint application, CC: Conventional care.


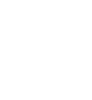

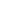

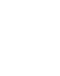


**Supplementary 9. Network plots**

9.1. The network plot for Overall Fatigue

Figure S9.1 The network plot for Overall Fatigue


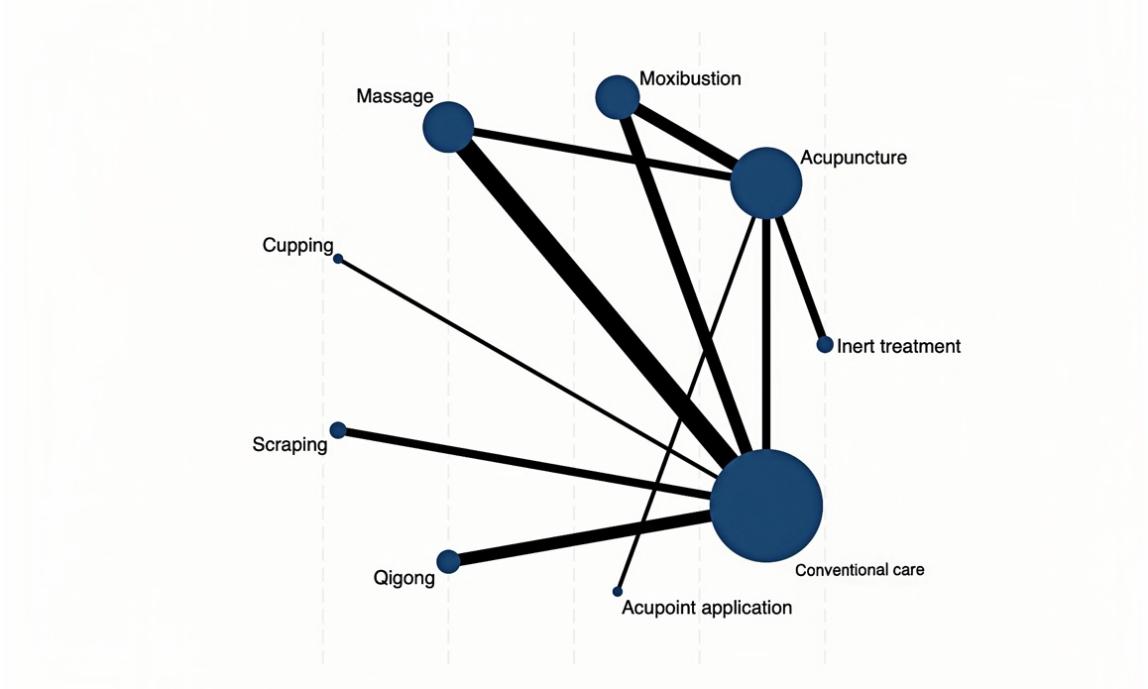


9.2. The network plot for Physical Fatigue

Figure S9.2 The network plot for Physical Fatigue


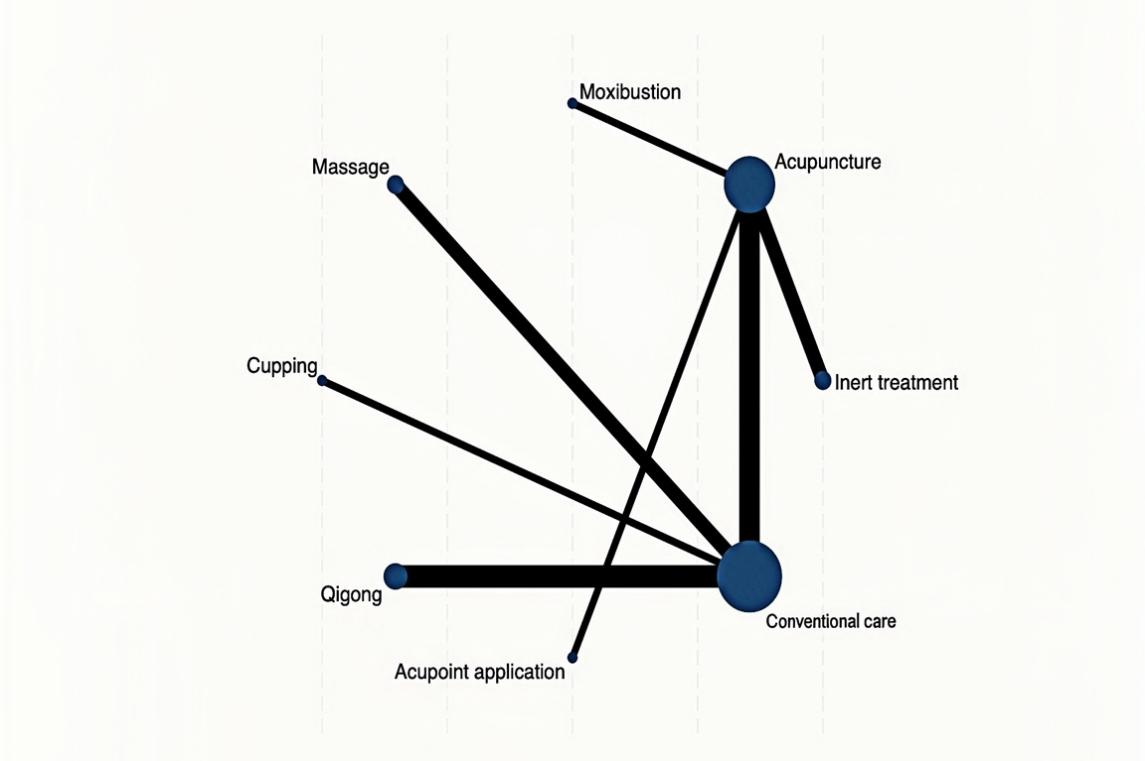


9.3. The network plot for Mental Fatigue

Figure S9.3 The network plot for Mental Fatigue


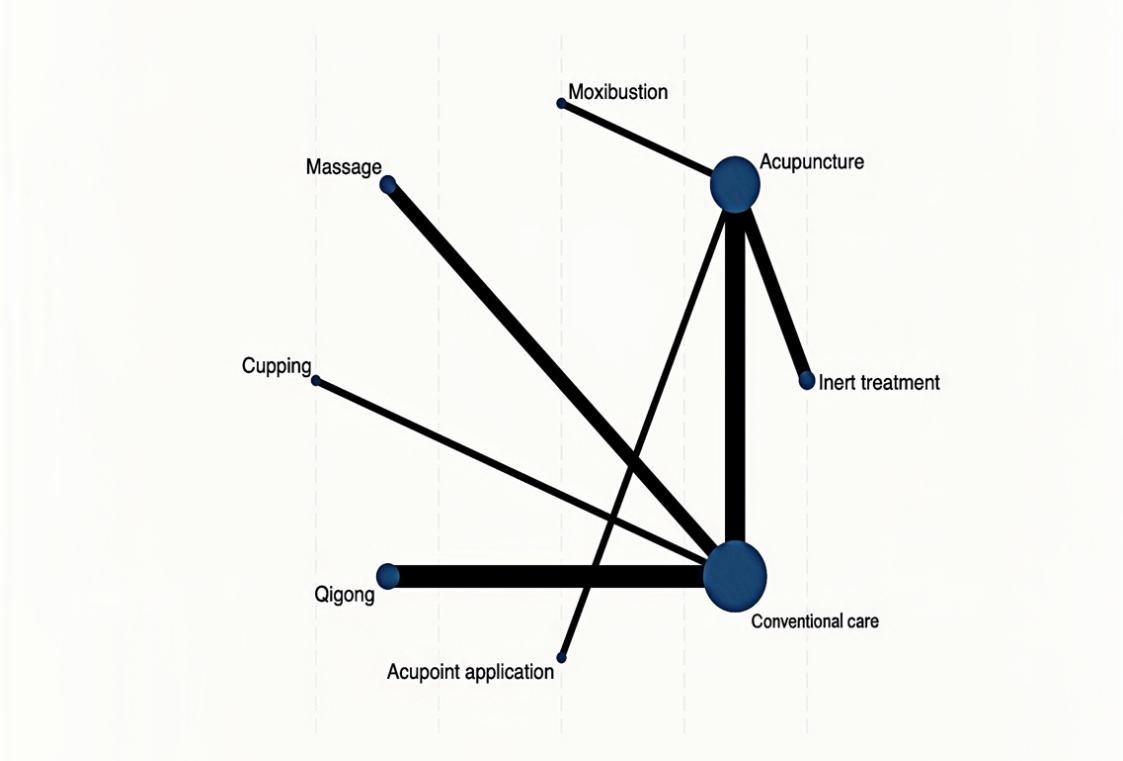


9.4. The network plot for Sleep quality

Figure S9.4 The network plot for Sleep quality


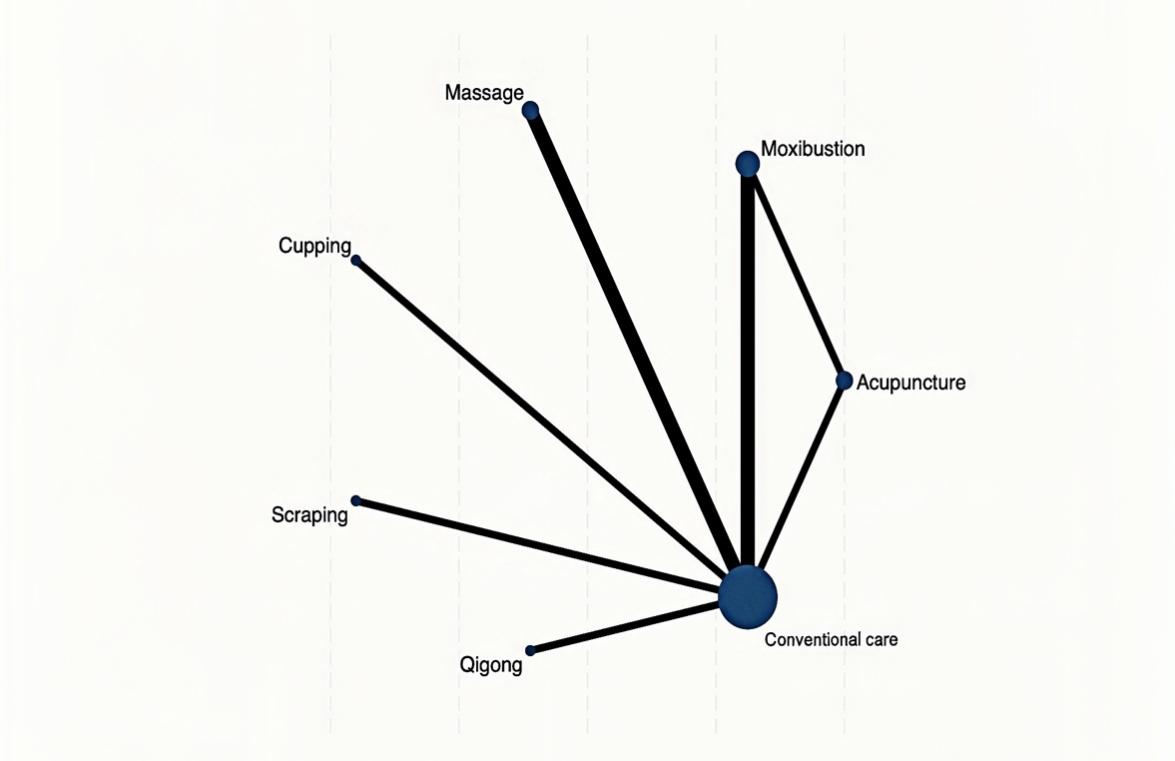


9.5. The network plot for Anxiety

Figure S9.5 The network plot for Anxiety


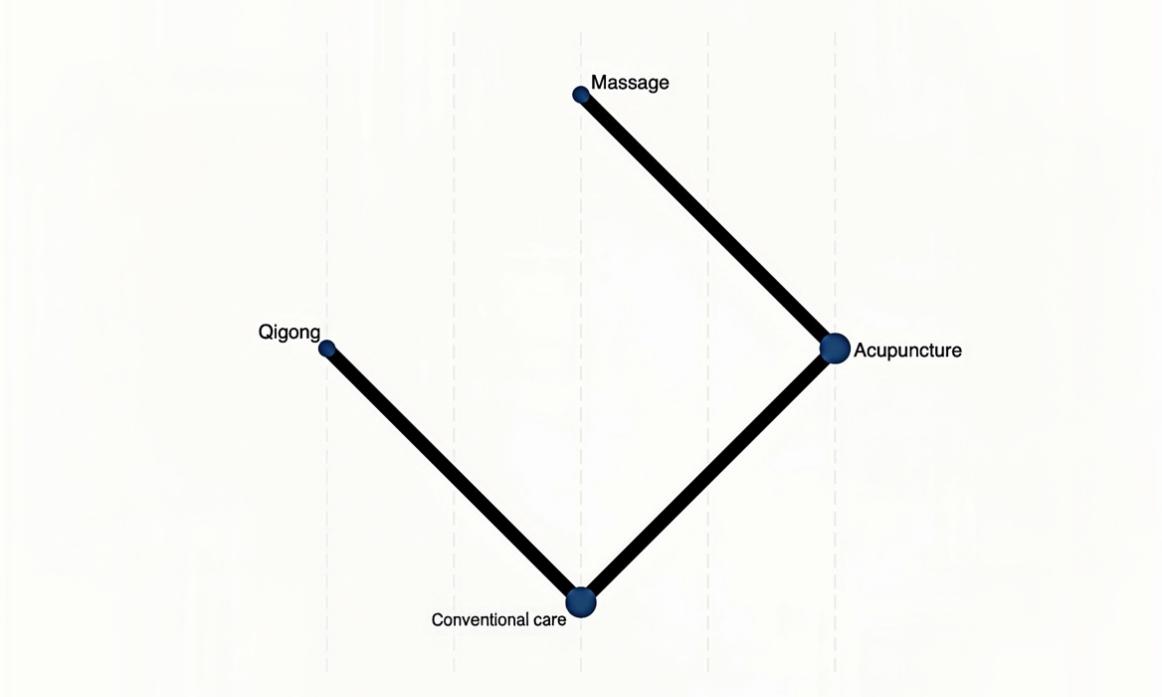


9.6. The network plot for Depression

Figure S9.6 The network plot for Depression

**
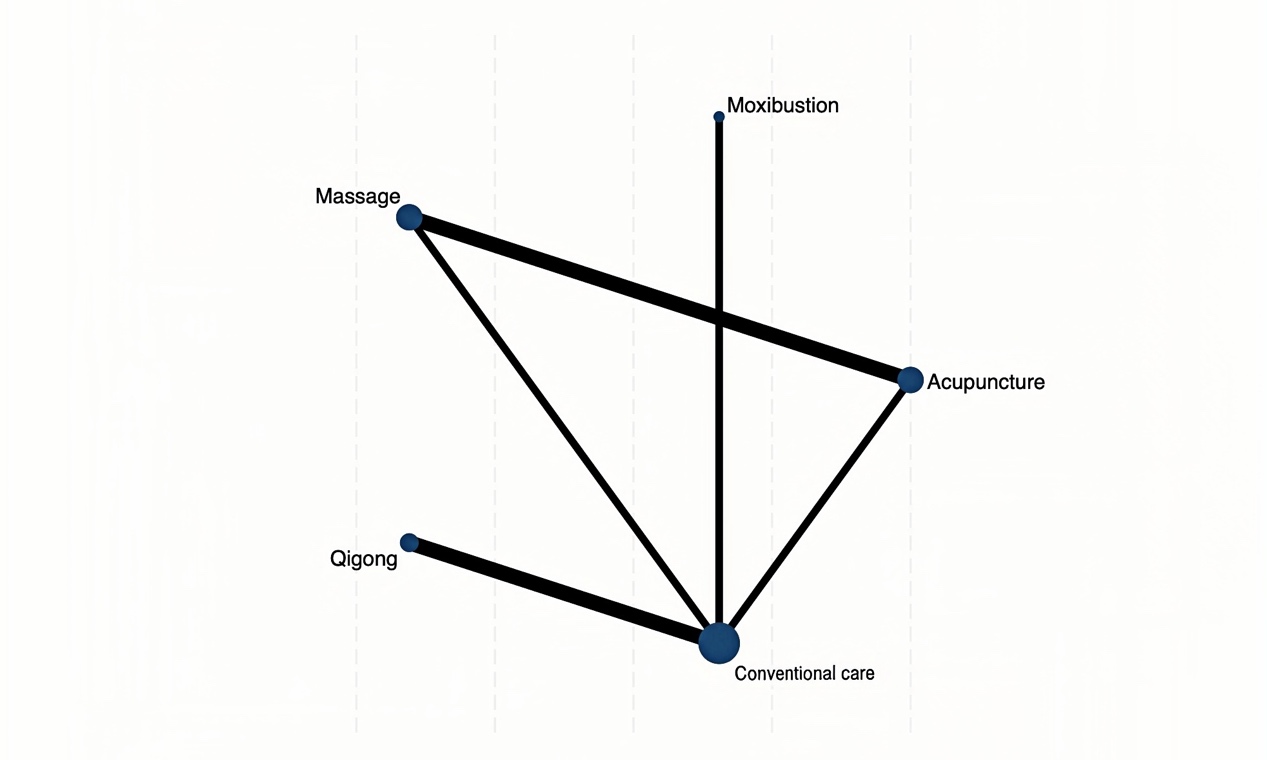
Supplementary 10. Rank results and SUCRA**

10.1. Rank results and SUCRA for Overall Fatigue

TableS10.1. The treatment ranking and SUCRA for Overall Fatigue

| Rank | Treatment node | SUCRA | PrBest | Mean rank |
| --- | --- | --- | --- | --- |
| **1^st^** | Moxibustion | 98.3 | 87.3 | 1.1 |
| **2^nd^** | Massage | 71.0 | 0.1 | 3.3 |
| **3^rd^** | Acupoint application | 70.6 | 9.7 | 3.4 |
| **4^th^** | Cupping | 51.8 | 2.6 | 4.9 |
| **5^th^** | Qigong | 50.1 | 0.0 | 5.0 |
| **6^th^** | Acupuncture | 46.2 | 0.0 | 5.3 |
| **7^th^** | Scraping | 45.0 | 0.3 | 5.4 |
| **8^th^** | Inert treatment | 12.7 | 0 | 0 |
| **9^th^** | Conventional care | 4.2 | 0 | 8.7 |


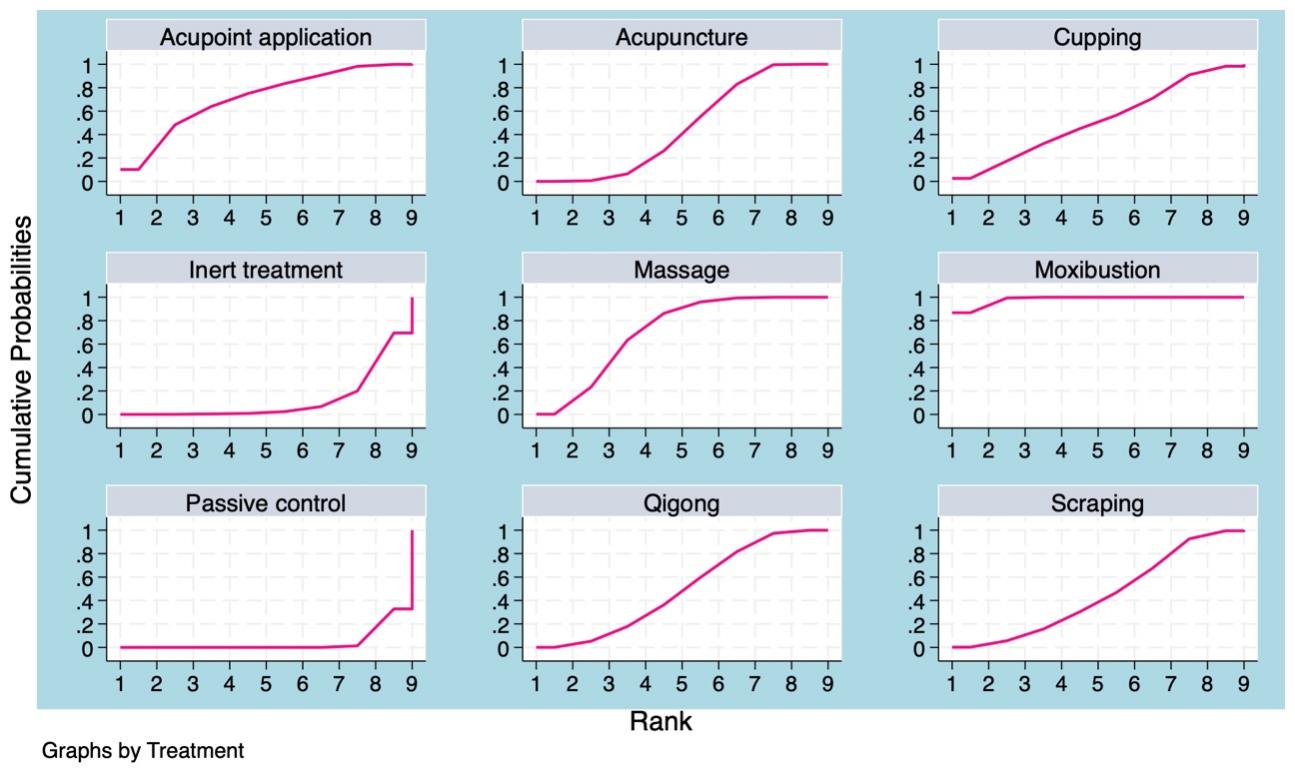


Abbreviations:IT:Inert treatment ,ACU:Acupuncture,MOX: Moxibustion, MAS:Massage, CUP: Cupping, SCR: Scraping QIG:Qigong, AA:Acupoint application, CC:Conventional care

10.2. Rank results and SUCRA for Physical Fatigue

TableS10.2. The treatment ranking and SUCRA for Physical Fatigue

| Rank | Treatment node | SUCRA | PrBest | Mean rank |
| --- | --- | --- | --- | --- |
| **1^st^** | Massage | 91.1 | 53.7 | 1.1 |
| **2^nd^** | Moxibustion | 87.7 | 37.2 | 1.9 |
| **3^rd^** | Acupoint application | 74.2 | 9.1 | 2.8 |
| **4^th^** | Acupuncture | 60.3 | 0.0 | 3.8 |
| **5^th^** | Inert treatment | 40.1 | 0.0 | 5.2 |
| **6^th^** | Qigong | 23.8 | 0.0 | 6.3 |
| **7^th^** | Cupping | 22.5 | 0 | 6.4 |
| **8^th^** | Conventional care | 0 | 0 | 8 |


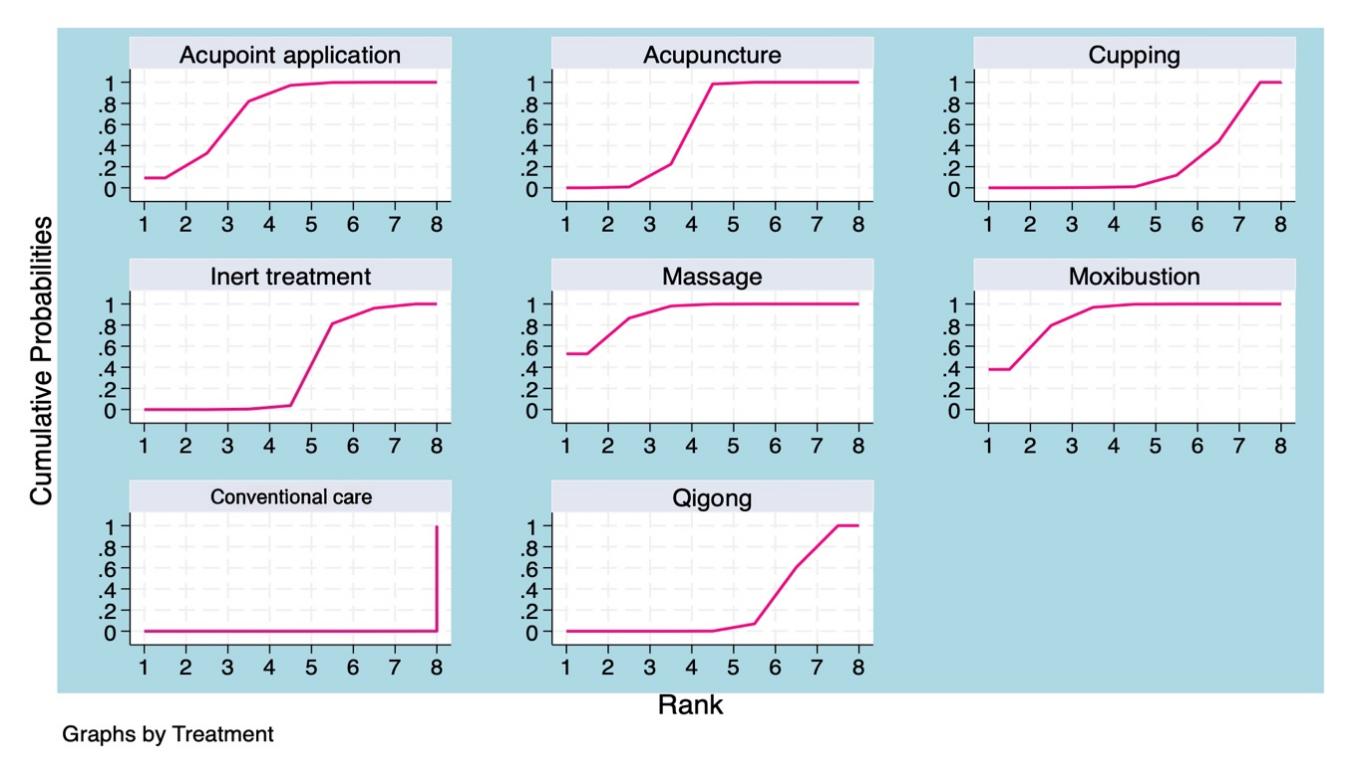


10.3. Rank results and SUCRA for Mental Fatigue

TableS10.3. The treatment ranking and SUCRA for Mental Fatigue

| Rank | Treatment node | SUCRA | PrBest | Mean rank |
| --- | --- | --- | --- | --- |
| **1^st^** | Massage | 88.7 | 54.0 | 1.8 |
| **2^nd^** | Moxibustion | 78.1 | 30.9 | 2.5 |
| **3^rd^** | Acupuncture | 62.5 | 1.0 | 3.6 |
| **4^th^** | Acupoint application | 61.3 | 12.0 | 3.7 |
| **5^th^** | Inert treatment | 38.2 | 0.9 | 5.3 |
| **6^th^** | Qigong | 34.4 | 0.1 | 5.6 |
| **7^th^** | Cupping | 27.7 | 1.1 | 6.1 |
| **8^th^** | Conventional care | 9.0 | 0.0 | 7.4 |


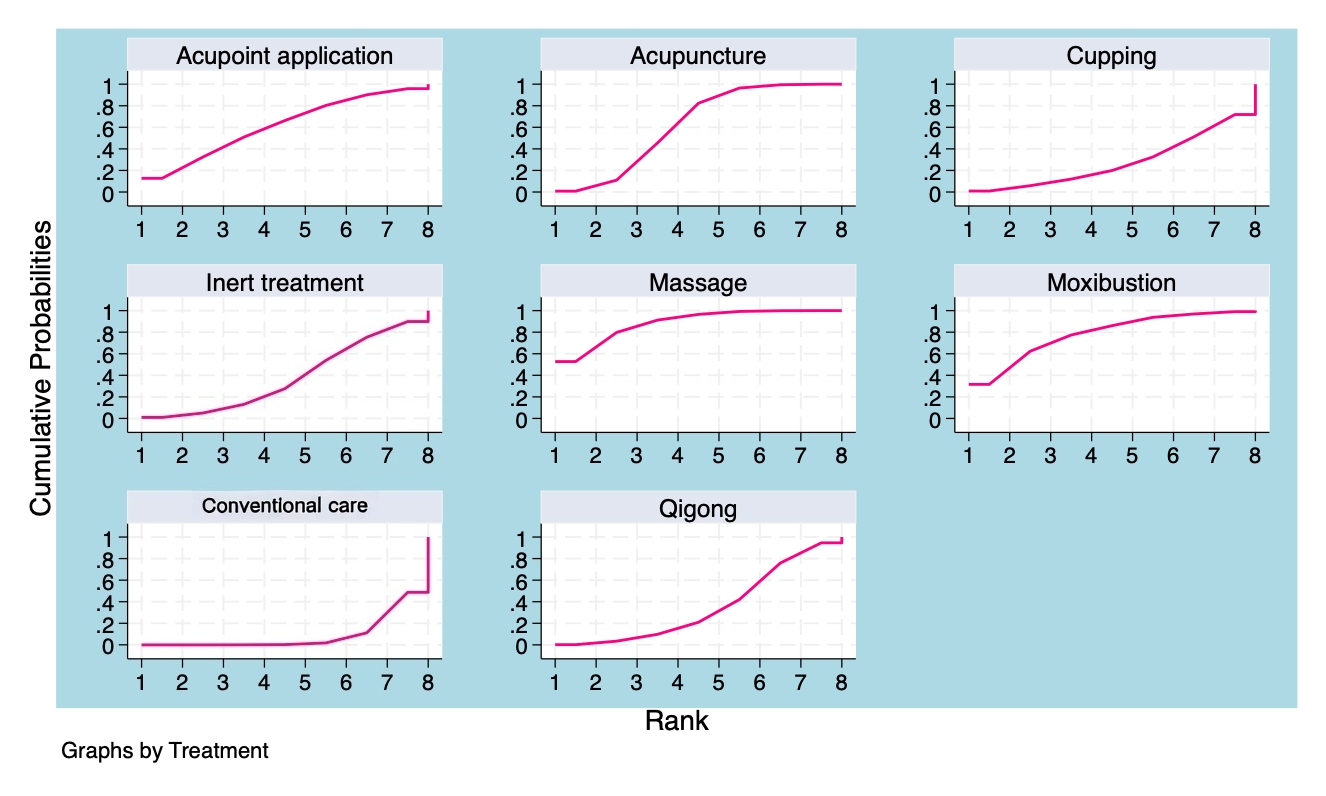


10.4. Rank results and SUCRA for Sleep quality

TableS10.4. The treatment ranking and SUCRA for Sleep quality

| Rank | Treatment node | SUCRA | PrBest | Mean rank |
| --- | --- | --- | --- | --- |
| **1^st^** | Cupping | 81.2 | 44.4 | 2.1 |
| **2^nd^** | Massage | 79.3 | 32.6 | 2.2 |
| **3^rd^** | Moxibustion | 72.7 | 14.8 | 2.6 |
| **4^th^** | Acupuncture | 61.8 | 7.7 | 3.3 |
| **5^th^** | Scraping | 28.0 | 0.4 | 5.3 |
| **6^th^** | Qigong | 22.0 | 0.2 | 5.7 |
| **7^th^** | Conventional care | 5.0 | 0.0 | 6.7 |


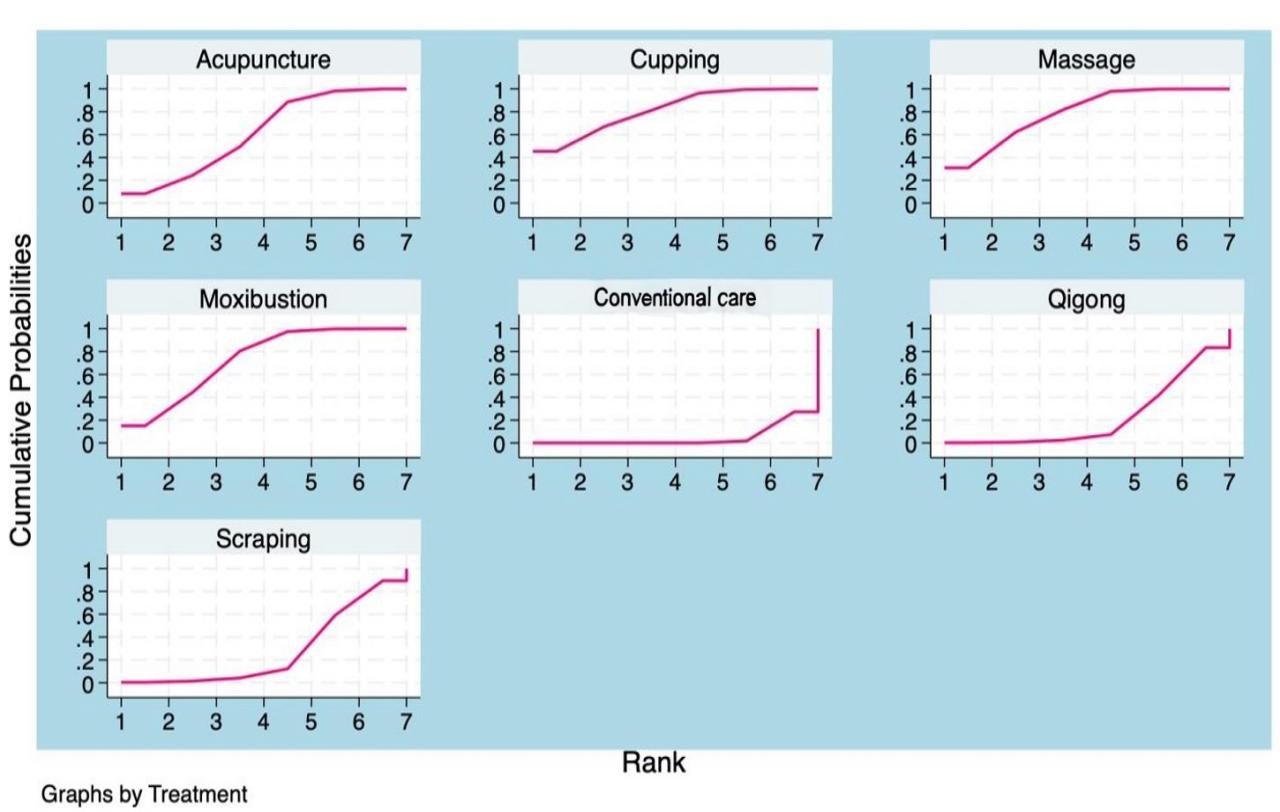


10.5. Rank results and SUCRA for Anxiety

TableS10.5. The treatment ranking and SUCRA for Anxiety

| Rank | Treatment node | SUCRA | PrBest | Mean rank |
| --- | --- | --- | --- | --- |
| **1^st^** | Massage | 92.0 | 82.7 | 1.2 |
| **2^nd^** | Acupuncture | 64.9 | 11.2 | 2.1 |
| **3^rd^** | Qigong | 26.9 | 5.6 | 3.2 |
| **4^th^** | Conventional care | 16.2 | 0.5 | 3.5 |


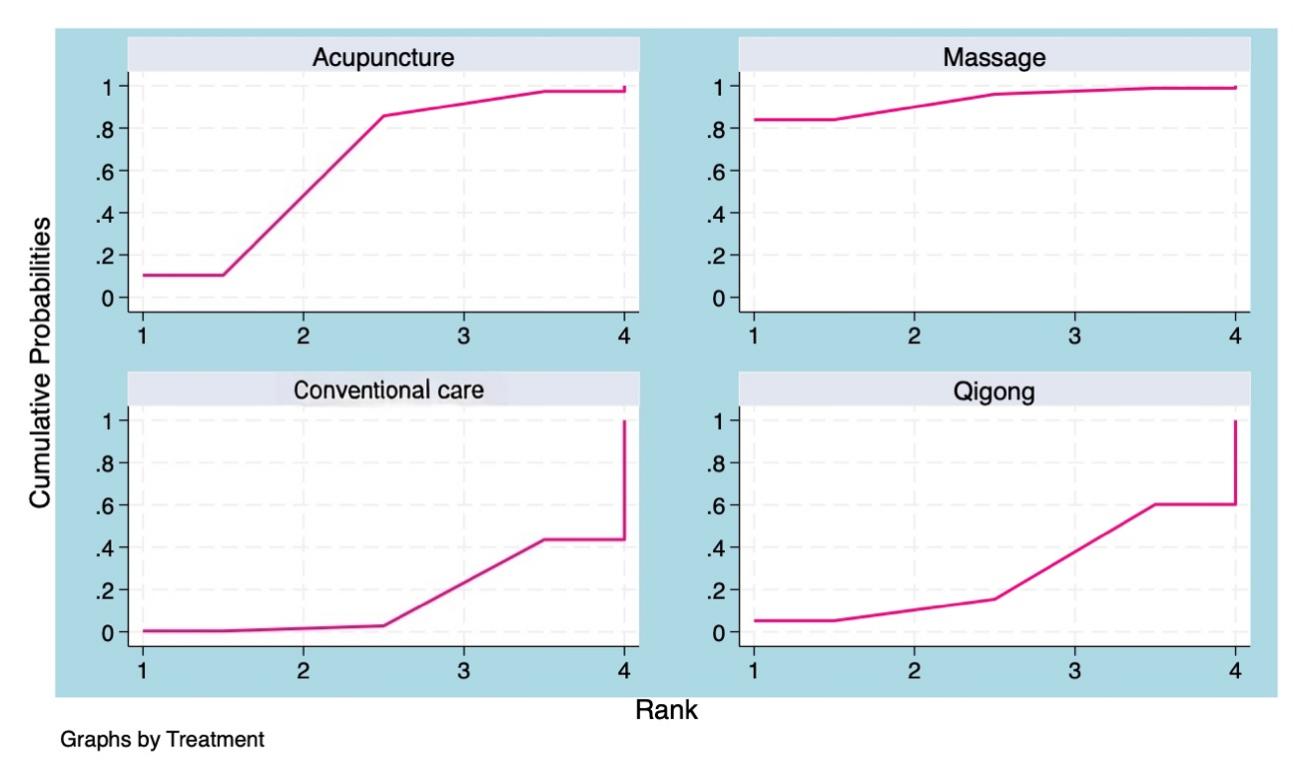


10.6. Rank results and SUCRA for Depression

TableS10.6. The treatment ranking and SUCRA for Depression

| Rank | Treatment node | SUCRA | PrBest | Mean rank |
| --- | --- | --- | --- | --- |
| **1^st^** | Massage | 97.0 | 89.6 | 1.1 |
| **2^nd^** | Acupuncture | 62.1 | 1.6 | 2.5 |
| **3^rd^** | Moxibustion | 53.9 | 7.9 | 2.8 |
| **4^th^** | Qigong | 36.9 | 0.9 | 3.5 |
| **5^th^** | Conventional care | 0.1 | 0.0 | 5.0 |


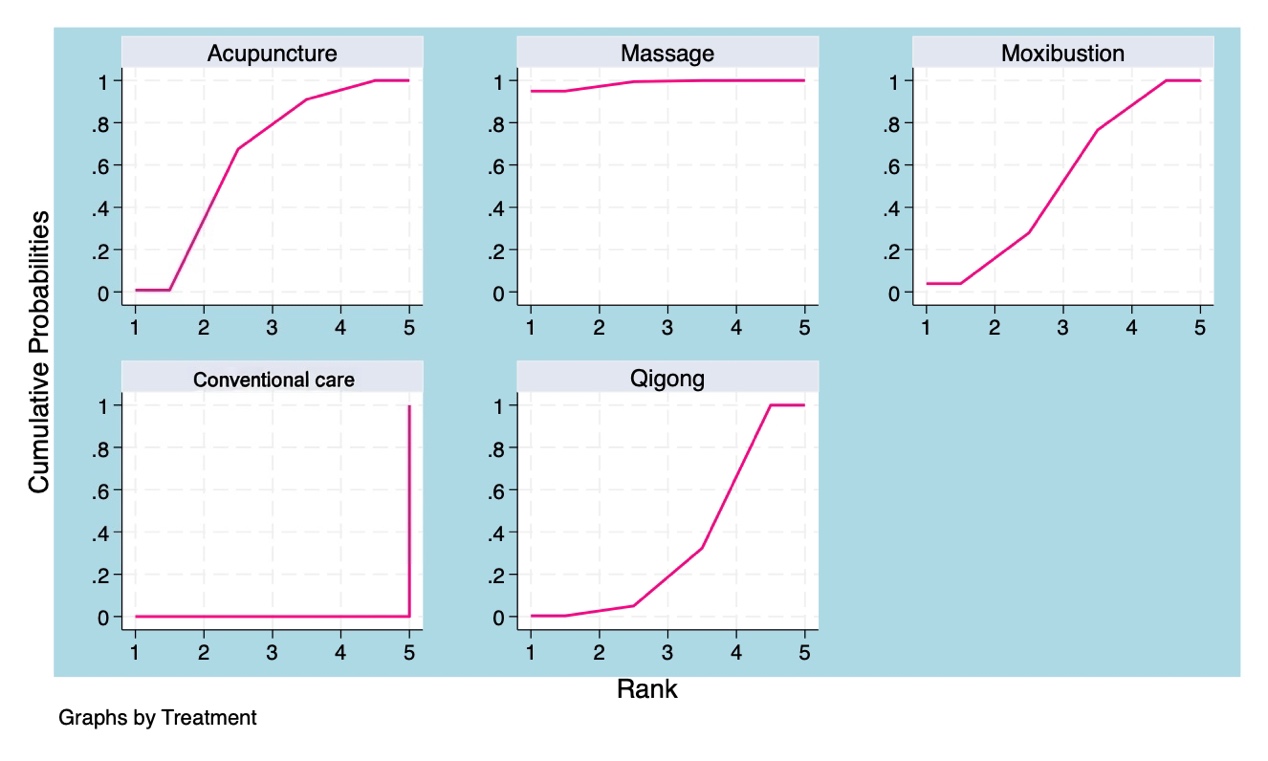


**Supplementary 11. League tables of the network meta-analysis**

11.1. The league table for Overall Fatigue

Table S11.1The league for Overall Fatigue

| **Inert treatment** |  |  |  |  |  |  |  |  |
| --- | --- | --- | --- | --- | --- | --- | --- | --- |
| **0.65**  **(0.03,1.28)** | **Acupuncture** |  |  |  |  |  |  |  |
| **1.66**  **(0.91,2.40)** | **1.00**  **(0.60,1.40)** | **Moxibustion** |  |  |  |  |  |  |
| **0.95 (**  **0.20,1.70)** | 0.30  (-0.12,0.72) | **-0.71**  **(-1.19,-0.22)** | **Massage** |  |  |  |  |  |
| 0.71  (-0.42,1.84) | 0.06  (-0.89,1.00) | -0.95  (-1.90,0.01) | -0.24  (-1.17,0.69) | **Cupping** |  |  |  |  |
| 0.62  (-0.34,1.57) | -0.04  (-0.76,0.68) | **-1.04**  **(-1.77,-0.31)** | -0.33  (-1.03,0.37) | -0.09  (-1.15,0.97) | **Scraping** |  |  |  |
| 0.68  (-0.18,1.55) | 0.03  (-0.57,0.63) | **-0.97**  **(-1.58,-0.36)** | -0.26  (-0.84,0.31) | -0.02  (-1.01,0.96) | 0.07  (-0.69,0.83) | **Qigong** |  |  |
| 1.04  (-0.02,2.10) | 0.39  (-0.46,1.24) | -0.61  (-1.56,0.33) | 0.09  (-0.86,1.04) | 0.33  (-0.94,1.61) | 0.43  (-0.69,1.54) | 0.36  (-0.68,1.40) | **Acupoint application** |  |
| **-0.19**  **(-0.92,0.54)** | -0.84  (-1.22,-0.46) | **-1.84**  **(-2.25,-1.44)** | **-1.14**  **(-1.48,-0.80)** | **-0.90 (**  **-1.76,-0.03)** | **-0.80**  **(-1.41,-0.19)** | **-0.87**  **(-1.33,-0.41)** | **-1.23**  **(-2.16,-0.29)** | **Conventional care** |

11.2. The league table for Physical Fatigue

Table S11.2 The league for Physical Fatigue

| **Inert treatment** |  |  |  |  |  |  |  |
| --- | --- | --- | --- | --- | --- | --- | --- |
| **0.36**  **(0.04,0.68)** | **Acupuncture** |  |  |  |  |  |  |
| **0.86**  **(0.24,1.47)** | 0.50  (-0.03,1.02) | **Moxibustion** |  |  |  |  |  |
| **0.94**  **(0.30,1.57)** | **0.58 (**  **0.03,1.12)** | 0.08  (-0.68,0.83) | **Massage** |  |  |  |  |
| -0.40  (-1.11,0.30) | **-0.77**  **(-1.39,-0.14)** | **-1.26**  **(-2.08,-0.44)** | **-1.34**  **(-2.04,-0.64)** | **Cupping** |  |  |  |
| **-0.34**  **(-0.85,0.16)** | **-0.71**  **(-1.11,-0.30)** | **-1.20**  **(-1.87,-0.54)** | **-1.28**  **(-1.80,-0.76)** | 0.06  (-0.54,0.66) | **Qigong** |  |  |
| 0.60  (-0.01,1.21) | 0.24  (-0.28,0.76) | -0.26  (-1.00,0.48) | -0.34  (-1.09,0.41) | **1.00**  **(0.19,1.82)** | **0.94**  **(0.28,1.60)** | **Acupoint application** |  |
| **-1.27**  **(-1.73,-0.82)** | **-1.63**  **(-1.95,-1.32)** | **-2.13**  **(-2.74,-1.52)** | **-2.21**  **(-2.65,-1.77)** | **-0.87**  **(-1.41,-0.33)** | **-0.93**  **(-1.20,-0.66)** | **-1.87**  **(-2.48,-1.26)** | **Conventional care** |

11.3. The league table for Mental Fatigue

Table S11.3The league for Mental Fatigue

| **Inert treatment** |  |  |  |  |  |  |  |
| --- | --- | --- | --- | --- | --- | --- | --- |
| 0.50  (-0.48,1.48) | **Acupuncture** |  |  |  |  |  |  |
| 1.02  (-0.69,2.74) | 0.52  (-0.88,1.93) | **Moxibustion** |  |  |  |  |  |
| 1.34  (-0.30,2.97) | 0.84  (-0.47,2.15) | 0.31  (-1.61,2.23) | **Massage** |  |  |  |  |
| -0.36  (-2.26,1.54) | -0.86  (-2.48,0.77) | -1.38  (-3.53,0.77) | -1.70  (-3.43,0.04) | **Cupping** |  |  |  |
| -0.14  (-1.64,1.36) | -0.64  (-1.78,0.49) | -1.17  (-2.98,0.64) | **-1.48**  **(-2.77,-0.19)** | 0.22  (-1.40,1.83) | **Qigong** |  |  |
| 0.56  (-1.15,2.27) | 0.06  (-1.34,1.47) | -0.46  (-2.45,1.53) | -0.77  (-2.69,1.15) | 0.92  (-1.23,3.07) | 0.71  (-1.10,2.51) | **Acupoint application** |  |
| -0.71  (-1.99,0.56) | **-1.21**  **(-2.02,-0.39)** | **-1.73**  **(-3.36,-0.11)** | **-2.05**  **(-3.07,-1.02)** | -0.35  (-1.76,1.06) | -0.57  (-1.36,0.22) | -1.27  (-2.90,0.35) | **Conventional care** |

11.4. The league table for Sleep quality

Table S11.4 The league for Sleep quality

| **Acupuncture** |  |  |  |  |  |  |
| --- | --- | --- | --- | --- | --- | --- |
| 0.47  (-1.39,2.32) | **Moxibustion** |  |  |  |  |  |
| 0.80  (-1.77,3.38) | 0.33  (-1.99,2.65) | **Massage** |  |  |  |  |
| 1.02  (-2.06,4.09) | 0.55  (-2.32,3.41) | 0.22  (-2.82,3.25) | **Cupping** |  |  |  |
| -2.11  (-5.15,0.92) | -2.58  (-5.40,0.24) | -2.91  (-5.90,0.07) | -3.13 (-6.56,0.30) | **Scraping** |  |  |
| -2.48  (-5.50,0.53) | **-2.95 (**  **-5.75,-0.15)** | **-3.28**  **(-6.25,-0.32)** | **-3.50**  **(-6.91,-0.09)** | -0.37  (-3.74,3.00) | **Qigong** |  |
| **-3.58**  **(-5.44,-1.73)** | **-4.05**  **(-5.53,-2.57)** | **-4.38**  **(-6.16,-2.60)** | **-4.60**  **(-7.05,-2.15)** | -1.47  (-3.87,0.93) | -1.10  (-3.47,1.27) | **Conventional care** |

11.5. The league table for Anxiety

Table S11.5 The league for Anxiety

| **Acupuncture** |  |  |  |
| --- | --- | --- | --- |
| 1.33  (-0.99,3.65) | **Massage** |  |  |
| -1.75  (-5.03,1.53) | -3.08  (-7.09,0.94) | **Qigong** |  |
| -2.02  (-4.36,0.32) | **-3.35**  **(-6.64,-0.05)** | -0.27  (-2.57,2.03) | **Conventional care** |

11.6. The league table for Depression

Table S11.6 The league for Depression

| **Acupuncture** |  |  |  |  |
| --- | --- | --- | --- | --- |
| -0.12  (-0.81,0.58) | **Moxibustion** |  |  |  |
| **0.38**  **(0.01,0.75)** | 0.50  (-0.22,1.22) | **Massage** |  |  |
| -0.28  (-0.87,0.31) | -0.16  (-0.73,0.41) | **-0.66**  **(-1.28,-0.04)** | **Qigong** |  |
| **-0.85**  **(-1.35,-0.34)** | **-0.73**  **(-1.21,-0.25)** | **-1.23**  **(-1.76,-0.69)** | **-0.57**  **(-0.88,-0.26)** | **Conventional care** |

**Supplementary 12. Sensitivity analyses for Overall Fatigue**

Table S12.a. Overall Fatigue removing studies with Invasive intervention

| **Moxibustion** |  |  |  |  |  |
| --- | --- | --- | --- | --- | --- |
| -1.06  (-1.60,-0.52) | **Massage** |  |  |  |  |
| -1.13  (-2.17,-0.10) | -0.07  (-1.07,0.92) | **Cupping** |  |  |  |
| -1.23  (-2.03,-0.42) | -0.17  (-0.92,0.58) | -0.09  (-1.22,1.03) | **Scraping** |  |  |
| -1.16  (-1.84,-0.47) | **-0.09**  **(-0.72,0.53)** | -0.02  (-1.06,1.02) | 0.07  (-0.74,0.89) | **Qigong** |  |
| **-2.03**  **(-2.51,-1.56)** | **-0.97**  **(-1.35,-0.59)** | **-0.90**  **(-1.82,0.02)** | **-0.80**  **(-1.45,-0.16)** | **-0.88**  **(-1.37,-0.38)** | **Conventional care** |

Table S12.b. Overall Fatigue studies removing studies with a sample size of≤30

| **Inert treatment** |  |  |  |  |  |
| --- | --- | --- | --- | --- | --- |
| 0.30  (-0.53,1.14) | **Acupuncture** |  |  |  |  |
| **1.22**  **(0.25,2.18)** | **0.91**  **(0.43,1.40)** | **Moxibustion** |  |  |  |
| 0.56  (-0.43,1.55) | 0.26  (-0.28,0.79) | **-0.65**  **(-1.23,-0.08)** | **Massage** |  |  |
| 0.34  (-0.73,1.40) | 0.03  (-0.63,0.70) | **-0.88**  **(-1.54,-0.22)** | **-0.23**  **(-0.82,0.37)** | **Qigong** |  |
| -0.54  (-1.50,0.43) | **-0.84**  **(-1.33,-0.35)** | **-1.75**  **(-2.24,-1.27)** | **-1.10**  **(-1.48,-0.72)** | **-0.87**  **(-1.33,-0.42)** | **Conventional care** |

Table S12.c. Overall Fatigue studies removing studies with studies with an intervention duration ≤4 weeks

| **Inert treatment** |  |  |  |  |  |  |  |  |
| --- | --- | --- | --- | --- | --- | --- | --- | --- |
| **0.65**  **(0.03,1.28)** | **Acupuncture** |  |  |  |  |  |  |  |
| **1.64**  **(0.89,2.40)** | **0.99 (0.58,1.41)** | **Moxibustion** |  |  |  |  |  |  |
| **1.07**  **(0.29,1.85)** | 0.42  (-0.05,0.88) | **-0.57**  **(-1.10,-0.05)** | **Massage** |  |  |  |  |  |
| 0.69  (-0.48,1.85) | 0.03  (-0.95,1.01) | -0.96  (-1.93,0.01) | -0.38  (-1.35,0.58) | **Cupping** |  |  |  |  |
| 0.59  (-0.39,1.58) | -0.06  (-0.82,0.70) | **-1.05**  **(-1.80,-0.31)** | -0.48  (-1.23,0.27) | -0.09  (-1.16,0.97) | **Scraping** |  |  |  |
| 0.66  (-0.24,1.56) | 0.01  (-0.64,0.65) | **-0.98**  **(-1.61,-0.36)** | -0.41  (-1.04,0.22) | -0.02  (-1.01,0.96) | 0.07  (-0.70,0.84) | **Qigong** |  |  |
| 1.04  (-0.02,2.10) | 0.39  (-0.47,1.24) | -0.60  (-1.55,0.35) | -0.03  (-1.00,0.95) | 0.36  (-0.94,1.66) | 0.45  (-0.70,1.59) | 0.38  (-0.69,1.45) | **Acupoint application** |  |
| -0.21  (-0.98,0.56) | **-0.86**  **(-1.32,-0.41)** | **-1.86**  **(-2.28,-1.43)** | **-1.28**  **(-1.71,-0.85)** | **-0.90**  **(-1.77,-0.03)** | **-0.80**  **(-1.42,-0.19)** | **-0.87**  **(-1.33,-0.41)** | **-1.25**  **(-2.22,-0.29)** | **Conventional care** |

**Supplementary 13. Grading the evidence using CINeMA for primary outcome**

**Table S13 CINEMA Assessments for Overall Fatigue**

| Comparison | Number of studies | Within-study bias | Reporting bias | Indirectness | Imprecision | Heterogeneity | Incoherence | Confidence rating |
| --- | --- | --- | --- | --- | --- | --- | --- | --- |
| A:B | 2 | Some concerns | Low risk | No concerns | No concerns | Some concerns | No concerns | Low |
| B:C | 3 | Major concerns | Low risk | No concerns | No concerns | No concerns | No concerns | Low |
| B:D | 2 | Major concerns | Low risk | No concerns | No concerns | Some concerns | No concerns | Very low |
| B:H | 1 | Major concerns | Low risk | No concerns | Some concerns | No concerns | No concerns | Very low |
| B:I | 2 | Major concerns | Low risk | No concerns | No concerns | No concerns | No concerns | Low |
| C:I | 3 | Major concerns | Low risk | No concerns | No concerns | No concerns | No concerns | Low |
| D:I | 5 | Major concerns | Low risk | No concerns | No concerns | No concerns | No concerns | Low |
| E:I | 1 | Major concerns | Low risk | No concerns | No concerns | Some concerns | No concerns | Very low |
| F:I | 2 | Some concerns | Low risk | No concerns | No concerns | Some concerns | No concerns | Low |
| G:I | 3 | Some concerns | Low risk | No concerns | No concerns | No concerns | No concerns | Moderate |
| A:C | 0 | Major concerns | Low risk | No concerns | No concerns | No concerns | No concerns | Low |
| A:D | 0 | Major concerns | Low risk | No concerns | No concerns | Some concerns | No concerns | Very low |
| A:E | 0 | Major concerns | Low risk | No concerns | Some concerns | No concerns | No concerns | Very low |
| A:F | 0 | Major concerns | Low risk | No concerns | Some concerns | No concerns | No concerns | Very low |
| A:G | 0 | Some concerns | Low risk | No concerns | Some concerns | No concerns | No concerns | Low |
| A:H | 0 | Major concerns | Low risk | No concerns | No concerns | Some concerns | No concerns | Very low |
| A:I | 0 | Major concerns | Low risk | No concerns | Some concerns | Some concerns | No concerns | Very low |
| B:E | 0 | Major concerns | Low risk | No concerns | Major concerns | No concerns | No concerns | Very low |
| B:F | 0 | Major concerns | Low risk | No concerns | No concerns | Major concerns | No concerns | Very low |
| B:G | 0 | Some concerns | Low risk | No concerns | No concerns | Major concerns | No concerns | Very low |
| C:D | 0 | Major concerns | Low risk | No concerns | No concerns | Some concerns | No concerns | Very low |
| C:E | 0 | Major concerns | Low risk | No concerns | No concerns | Some concerns | No concerns | Very low |
| C:F | 0 | Major concerns | Low risk | No concerns | No concerns | Some concerns | No concerns | Very low |
| C:G | 0 | Some concerns | Low risk | No concerns | No concerns | Some concerns | No concerns | Low |
| C:H | 0 | Major concerns | Low risk | No concerns | Some concerns | No concerns | No concerns | Very low |
| D:E | 0 | Major concerns | Low risk | No concerns | Some concerns | Some concerns | No concerns | Very low |
| D:F | 0 | Major concerns | Low risk | No concerns | Some concerns | No concerns | No concerns | Very low |
| D:G | 0 | Some concerns | Low risk | No concerns | Some concerns | No concerns | No concerns | Low |
| D:H | 0 | Major concerns | Low risk | No concerns | Major concerns | No concerns | No concerns | Very low |

Note: Treatment: A="Inert treatment" B="Acupuncture" C="Moxibustion" D="Massage" E="Cupping" F="Scraping" G="Qigong" H="Acupoint application" I="Conventional care", The “number of studies” column entries with “0” indicate that no head-to-head trials were available for that comparison.
